# The Origin of Broad Emission in ⟨100⟩ Two-Dimensional Perovskites: Extrinsic vs Intrinsic Processes

**DOI:** 10.1021/acsenergylett.2c02123

**Published:** 2022-10-31

**Authors:** Simon Kahmann, Daniele Meggiolaro, Luca Gregori, Eelco K. Tekelenburg, Matteo Pitaro, Samuel D. Stranks, Filippo De Angelis, Maria A. Loi

**Affiliations:** † Photophysics and OptoElectronics Group, Zernike Institute for Advanced Materials, University of Groningen, Nijenborgh 4, 9747 AG Groningen, The Netherlands; ‡ Cavendish Laboratory, University of Cambridge, JJ Thomson Avenue CB3 0HE Cambridge, U.K.; § Computational Laboratory for Hybrid/Organic Photovoltaics (CLHYO), Istituto CNR di Scienze e Tecnologie Chimiche (SCITEC−CNR), Via Elce di Sotto 8, 06123 Perugia, Italy; ∥ Department of Chemistry, Biology and Biotechnology, University of Perugia, Via Elce di Sotto 8, 06123 Perugia, Italy; ⊥ Department of Chemical Engineering and Biotechnology, University of Cambridge, Philippa Fawcett Drive CB3 0AS Cambridge, U.K.; # Department of Natural Sciences & Mathematics, College of Sciences & Human Studies, Prince Mohammad Bin Fahd University, Dhahran 34754, Saudi Arabia

## Abstract

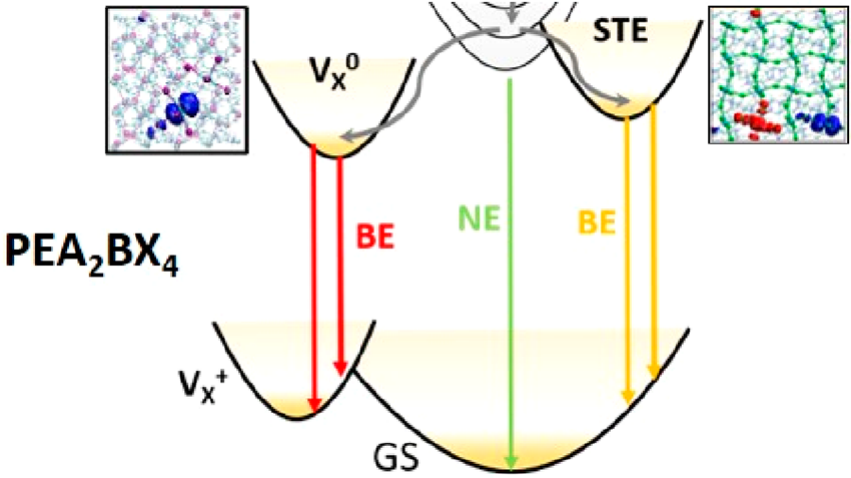

2D metal halide perovskites can show narrow and broad emission bands (BEs), and the latter’s origin is hotly debated. A widespread opinion assigns BEs to the recombination of intrinsic self-trapped excitons (STEs), whereas recent studies indicate they can have an extrinsic defect-related origin. Here, we carry out a combined experimental–computational study into the microscopic origin of BEs for a series of prototypical phenylethylammonium-based 2D perovskites, comprising different metals (Pb, Sn) and halides (I, Br, Cl). Photoluminescence spectroscopy reveals that all of the compounds exhibit BEs. Where not observable at room temperature, the BE signature emerges upon cooling. By means of DFT calculations, we demonstrate that emission from halide vacancies is compatible with the experimentally observed features. Emission from STEs may only contribute to the BE in the wide-band-gap Br- and Cl-based compounds. Our work paves the way toward a complete understanding of broad emission bands in halide perovskites that will facilitate the fabrication of efficient narrow and white light emitting devices.

Metal halide perovskites and perovskite-inspired materials are excellent candidates for optoelectronic applications.^1,2^ In addition to their cheap and straightforward fabrication from solution, compositional and dimensional engineering work as powerful levers to tune their optoelectronic properties. Low-dimensional halide perovskites are generally based on metal halide octahedra, whose interaction is limited by long and bulky organic spacer molecules.^3,4^ This imposes a quantum and a dielectric confinement onto charge carriers and results in large exciton binding energies.^5^ Such strongly bound excitons exhibit high luminescence quantum yields, rendering low-dimensional halide perovskites excellent candidates for use in light-emitting diodes.^6^

Whereas the narrow luminescence line width of many 2D perovskites is generally attributed to the radiative decay of free excitons,^7^ most 1D and 0D compounds exhibit broad emission, strongly red shifted from the absorption onset, ascribed to recombination of self-trapped excitons (STEs).^8−11^ Such broad emission bands (BEs) are also present in some 2D perovskites, where they seem to solely dominate the emission of the ⟨110⟩ family.^12^ It has thus become widespread in the field to attribute broad emission bands in halide perovskites and perovskite-inspired compounds to the formation of self-trapped excitons.

A curious case is the family of ⟨100⟩ 2D perovskites, in which slabs of corner-sharing metal halide octahedra are sandwiched between layers of spacer molecules. Their typical narrow and bright emission from free excitons is occasionally accompanied by a red-shifted broad emission band—in particular in single crystals and at low temperature, as well as in strongly distorted compounds.^13−18^ Here, the origin of the BE is hotly debated. Still often also attributed to STEs,^12^ recent reports have questioned this assertion.^13,14,19^ In particular, some of us argued that halide-related defects were responsible for the BE, since a large sample-to-sample variation was observed in single crystals, and the red-shifted emission could be excited through photons of energy below the band gap.^13^ Similarly, Yin et al.^19^ and Zhang et al.^14^ synthesized single crystals and observed a variation of PL spectra, which they attributed to iodide-related defects—vacancies in the former and interstitials in the latter case. Interstitials have furthermore been invoked by Booker et al. for a related PbI-based compound.^15^ Moreover, Yu et al. studied Sn-doped PEA_2_PbI_4_ and attributed the formation of pronounced low-energy luminescence to extrinsic STEs.^20^ A shift in opinion thus seemingly excludes the classical STE hypothesis for ⟨100⟩ compounds. However, although the works by Yin, Zhang, Yu, and us assume an extrinsic origin, the actual origin of the emission remains unclear. In addition, most studies have so far relied on one single compound, such as the prototypical PEA_2_PbI_4_, and the impact of the metal or the halide constituents on the BE remains unexplored.

In this work we provide new insights into the origin of broad emission bands in ⟨100⟩ compounds by conducting a joint experimental and computational study. Optical experiments carried out on the prototypical phenylethylammonium-based (PEA) family, including PEA_2_PbCl_4_, PEA_2_PbBr_4_, PEA_2_PbI_4_, and PEA_2_SnI_4_, reveal that all of the compounds exhibit BEs. Where not observable at room temperature, the BE signature emerges upon cooling. In parallel, we conducted a density functional theory (DFT) study to investigate the recombination of charge carriers via self-trapping and via native defects. DFT simulations show that in the wide-gap PEA_2_PbBr_4_ and PEA_2_PbCl_4_ phases, excitons can self-trap, but into a shallow state. In contrast, no evidence for carrier self-trapping in PEA_2_PbI_4_ and PEA_2_SnI_4_ could be found. Defect calculations, on the other hand, identify halide vacancies for all cases to result in optical transitions, in excellent agreement with experiments. Such defects occur at very low densities in these perovskites under thermodynamic equilibrium, suggesting that their formation is driven by kinetic factors during material synthesis.

We fabricated a set of two-dimensional halide perovskite thin films based on the prototypical spacer cation phenylethylammonium and studied their photoluminescence (upon excitation at 3.1 or 4.6 eV). As shown in Figure 1a, substituting the metal and the halide constituents shifts the bright and narrow luminescence of these compounds from the blue (PEA_2_PbBr_4_) into the green (PEA_2_PbI_4_) or the red (PEA_2_SnI_4_) spectral region.^21^ Luminescence of the free exciton transition can be observed at 3.02, 2.38, and 1.98 eV, respectively. None of the three compounds exhibit any strong BE at room temperature. The less studied variant PEA_2_PbCl_4_ shows a different picture. Following the trend of widening band gap for smaller halides, its free exciton transition lies in the ultraviolet region around (340 nm, 3.64 eV; absorbance spectra are provided in Figure S1 in the Supporting Information),^15^ while a broad emission band covers the entire visible spectral range. Thus, PEA_2_PbCl_4_ emits white light upon UV excitation (Figure S1).

**Figure 1:**
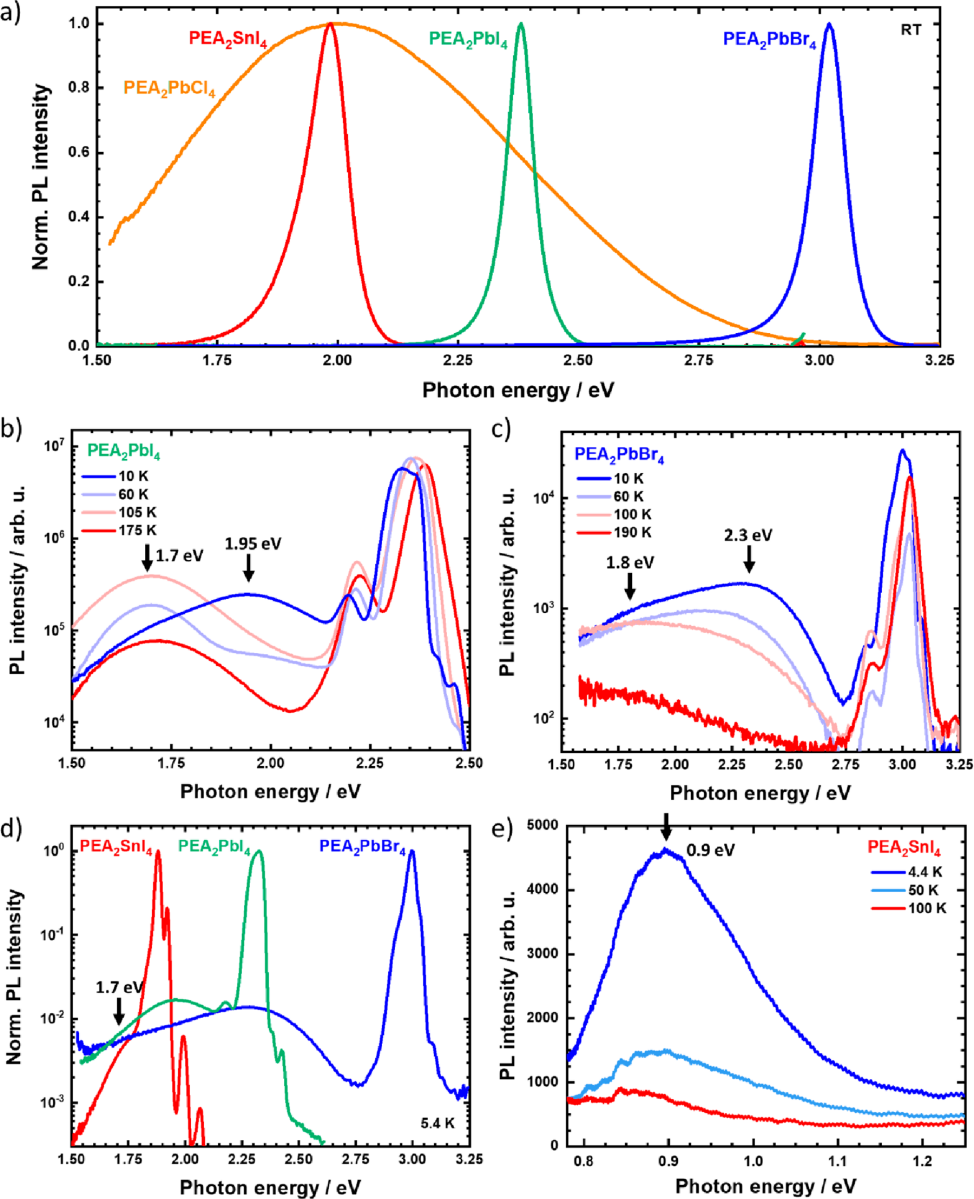
Photoluminescence spectra of the studied compounds under different conditions (excitation at 4.6 eV for PEA_2_PbCl_4_; 3.1 eV for the others). (a) Room-temperature spectra in the visible range on a linear scale. Semilogarithmic spectra of PEA_2_PbI_4_ (b) and PEA_2_PbBr_4_ (c) upon temperature variation. The black arrows indicate the peak energy of the emerging broad emission bands. (d) Comparison of PEA_2_PbBr_4_, PEA_2_PbI_4_, and PEA_2_SnI_4_ PL at 5.4 K indicating the pronounced red-shifted BE in all cases. (e) PL of PEA_2_SnI_4_ in the near-infrared spectral region upon temperature variation. The arrow identifies an additional BE at 0.9 eV.

Previous studies highlighted a pronounced BE of PEA_2_PbI_4_ for single crystals.^13,14,19^ Where not observable at room temperature, the BE still emerged when the material was cooled.^13^ We thus performed temperature-dependent PL spectroscopy, as shown in Figure 1b,c for PEA_2_PbI_4_ and PEA_2_PbBr_4_ thin films. Broad and red-shifted emission bands form in both cases and are clearly observable below 200 K. PEA_2_PbI_4_ exhibits two broad but distinct bands around 1.7 and 1.95 eV, the latter of which becomes prominent below 60 K. Transitions for the bromide variant in Figure 1c overlap more strongly, but a red-shifted emission centered around 1.8 eV below 200 K clearly shifts toward 2.3 eV upon further cooling. We note that the intricate substructure of the narrow emission was discussed previously.^21^

PEA_2_SnI_4_ behaves differently. Figure 1d shows its spectrum at 5.4 K in addition to those of PEA_2_PbI_4_ and PEA_2_PbBr_4_ to highlight the absence of a pronounced red-shifted band distinct from the narrow emission of the free exciton. We merely identify a broader background around 1.7 eV merging with the narrow emission. To the best of our knowledge, there has been only one report on of a BE comparable to that of lead-based variants for systems based on tin.^22^ However, when deliberately exploring much lower energy, corresponding to the near-infrared spectral region, we find that cooling gives rise to the formation of a broad signal at 0.9 eV (Figure 1e), which has so far not been reported and which scales sublinearly with incident fluence (Figure S2).

In summary, we note that all compounds exhibit broad and red-shifted emission bands (see Table 1). Whereas PEA_2_PbCl_4_ already exhibits strong white luminescence at room temperature, PEA_2_PbBr_4_, PEA_2_PbI_4_, and PEA_2_SnI_4_ reveal increasingly pronounced BEs at low temperature.

**Table 1:** Predicted Band Gaps, (0/−) and (+/0) Transitions of STEs, (+/0) Transitions of Halogen Vacancies V_X_ (Equatorial, eq; Apical, ap), and Calculated PL Emission Energies of STE and V_X_^a^

Phase	TIL/eV	PL emission theory/eV	PL emission experiments/eV
PEA_2_SnI_4_ (*E*_g_ = 2.26 eV)			
STE			
V_I_ eq	(+/0)/1.42	0.82	0.9
V_I_ ap	(+/0)/1.84	1.75	1.7
PEA_2_PbI_4_ (*E*_g_ = 2.58 eV)			
STE			
V_I_ eq	(+/0)/1.94	1.27	(1.7)
V_I_ ap	(+/0)/2.07	1.97	1.95
PEA_2_PbBr_4_ (*E*_g_ = 3.26 eV)			
STE	(0/−)/3.48	2.19	2.3
	(+/0)/0.05^b^		
V_Br_ eq	(+/0)/2.56	1.78	1.75
V_Br_ ap	(+/0)/2.93	1.73	
PEA_2_PbCl_4_ (*E*_g_ = 3.91 eV)			
STE	(0/−)/3.88	1.89	2
	(+/0)/0.12^b^		
V_Cl_ eq	(+/0)/2.89	1.97	
V_Cl_ ap	(+/0)/3.29	1.85	

a All values were calculated at the PBE0 level by correcting for SOC (see Computational Details). In the last column the experimentally observed transitions for the four perovskites are reported for comparison.

b The (+/0) transitions associated with the self-trapping of the hole are calculated at the PBE0 without SOC corrections, due to the limited influence of SOC on the VB.

We investigated the microscopic origin of the BEs by DFT by simulating two radiative pathways that possibly result in subgap emission in these materials: i.e. (i) the radiative emission from STEs and (ii) the radiative decay of trapped holes/electrons at point defects.

A quantitative description of these processes requires the use of advanced DFT techniques able to accurately predict the electronic properties of the perovskites for a correct estimate of the charge transition levels.^23^ The computational approach applied in this work closely follows that validated for defect calculations in 3D perovskites^24−26^ based on the use of the hybrid PBE0 functional^27^ with the inclusion of spin–orbit coupling (SOC). As discussed in the Supporting Information and in agreement with previous works in the literature,^28,29^ this approach provides an accurate description of the electronic properties of 2D perovskites, predicting band gaps in good agreement with G_0_W_0_ calculations and experiments. Due to the limited influence on the VBM states, SOC corrections have been included only in the calculation of ionization levels and emission energies of CB-related defects (see details in Computational Details).

The self-trapping of photogenerated charge carriers has been simulated by performing ion geometry relaxations of the pristine perovskites’ supercells by constraining one electron and one hole in the CB and in the VB, respectively, by imposing the triplet state. By this approach the lowest energy configuration of the lattice in response to the parallel injection of one hole and one electron is predicted, which is a fair approximation to the electron–hole pair relaxation process after photoexcitation: i.e., the STE.

We found very limited lattice relaxations and no evidence of charge carrier localization in PEA_2_SnI_4_ and PEA_2_PbI_4_ perovskites (see Figure 2a,b), indicating that in these systems the self-trapping of charge carriers to form the STE can be excluded. On the other hand, for the PEA_2_PbBr_4_ and PEA_2_PbCl_4_ phases a clear localization of the hole/electron is observed on different PbBr_6_ and PbCl_6_ octahedra (see Figure 2c,d). This localization entails a shortening/elongation of the Pb–halide bonds with stabilization energies of the electron–hole couples of 0.23 and 0.45 eV for PEA_2_PbBr_4_ and PEA_2_PbCl_4_, respectively (at the PBE0 level without SOC corrections). In order to provide a more detailed analysis of the process, the thermodynamics of trapping of the electron and the hole has been studied independently by calculating the (0/−) and (+/0) transitions, associated with the localization of the electron and the hole, respectively, by relaxing the lattice in the −1 and +1 charged states. Due to the dramatic effects induced by SOC on the Pb p orbitals, the (0/−) transitions were calculated at the PBE0-SOC level of theory. The results, reported in Table 1, show that both the (0/−) and the (+/0) transitions occur close to or within the band edges of the materials, highlighting a low tendency of charge carriers to be self-trapped in the perfect lattice. Specifically, while the trapping of the electron and hole is thermodynamically slightly favored in PEA_2_PbCl_4_, by showing (+/0) and (0/−) transitions above and below the VB and CB, respectively, the trapping of the electron to form the STE is unfavorable in PEA_2_PbBr_4_ (the (0/−) transition is placed at 0.22 eV above the CB). The predicted vertical transitions associated with STE emission (correcting for SOC effects on the (0/−) transition) amount to 2.19 and 1.89 eV for PEA_2_PbBr_4_ and PEA_2_PbCl_4_, respectively, and are close in energy to the BEs peaking at 2.3 and 2 eV (see Figure 1a and Table 1). These results indicate that STE emission can likely contribute to BEs only in PEA_2_PbCl_4_, whereas emission from defect states is expected to dominate subgap emission in the remaining compounds. This correlates with the observation of pronounced BE from PEA_2_PbCl_4_ already at room temperature, where the other thin films exhibit negligible BE intensity.

**Figure 2:**
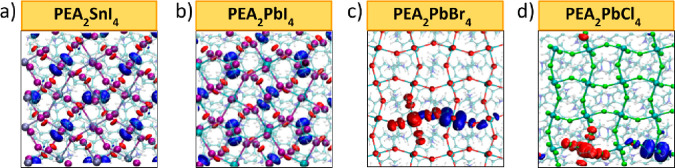
Plots of the hole (red) and electron (blue) Kohn–Sham (KS) levels for PEA_2_SnI_4_ (a), PEA_2_PbI_4_ (b), PEA_2_PbBr_4_ (c), and (d) PEA_2_PbCl_4_ after relaxing in the triplet state (orbital plot isovalue 0.03 au^–3/2^). Only PEA_2_PbBr_4_ and PEA_2_PbCl_4_ show a localization of the charge carriers in local lattice distortions, indicating the formation of shallow STEs.

In order to investigate possible extrinsic origins of the BEs, we first studied the nature and the trapping activity of the most stable point defects in PEA_2_SnI_4_ and PEA_2_PbI_4_. The defect formation energies (DFEs) and the thermodynamic ionization levels (TILs) of PEA_2_PbI_4_ and PEA_2_SnI_4_ are reported in Figure 3. In PEA_2_PbI_4_ the most stable defects are iodine vacancies (V_I_^+^), PEA vacancies (V_PEA_^–^), and iodine interstitials (I_i_^0^), which pin the Fermi level of the system close to the middle of the band gap (as indicated by a vertical dashed line in Figure 3a). For PEA_2_SnI_4_ an increased stability of the acceptor defects compared to the lead analogue is reported, with V_I_^+^, V_PEA_^–^, and V_Sn_^2–^ defects pinning the Fermi level at ∼0.6 eV above the VB, indicating a moderate p-doping.

**Figure 3:**
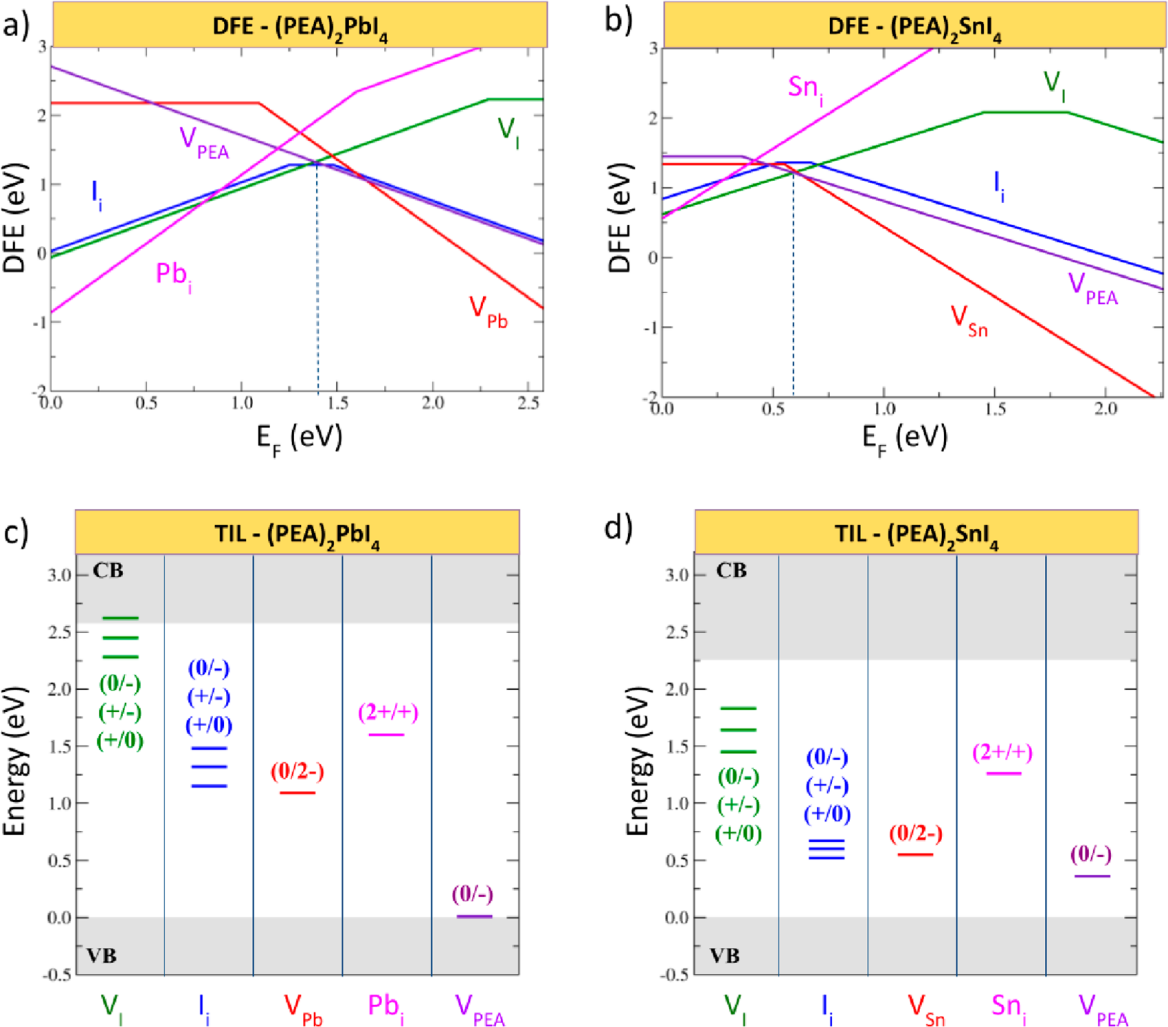
(a, b) Defect formation energies (DFE) under I-medium condition and (c, d) thermodynamic ionization levels (TILs) of native point defects in PEA_2_PbI_4_ and PEA_2_SnI_4_, calculated at the PBE0 level. The band gaps in the diagrams correspond to those calculated at the PBE0-SOC level of theory. The dashed lines indicate the position of the native Fermi level.

V_I_ are the dominating defects in the perovskites and may occur in two different positions within the inorganic layers: i.e., apical and equatorial (see Figure 4a,b). Both are stable in the positive +1 form at the native Fermi level and show a comparable stability, with relative energies differing by ∼0.2 eV at most. The plots in Figure 3 contain the results for the equatorial vacancies. The equatorial V_I_^+^ shows deep (+/0) transitions placed at 2.29 and 1.45 eV (1.94 and 1.42 eV by including SOC) above the VBM for lead and tin perovskites, respectively. On the other hand, apical V_I_^+^ show (+/0) transitions that are higher in energy and placed at ∼0.4 eV below the CBM in both cases. The analysis of the Kohn–Sham (KS) orbitals associated with the trapped electrons on the defect site confirms a strong localization in the case of the equatorial vacancy and a weaker localization in apical position (see Figure 4f,g), in agreement with the study of Yin et al.^19^ Notably, the deep (electron) trapping nature of equatorial V_I_ in PEA_2_PbI_4_ is in stark contrast with the shallow nature of this defect in the 3D MAPbI_3_.^25^ This change is readily understood by observing that quantum confinement in 2D perovskites leads to an opening of the band gap through a downshift (upshift) of the VB (CB) compared to the 3D phase (see Figures S4 and S5 in the Supporting Information). The upshift of the CB leads to a deepening of the (+/0) transition and to the emergence of populated Pb p orbital states localized on undercoordinated Pb ions on the neutral vacancy defect site.

**Figure 4:**
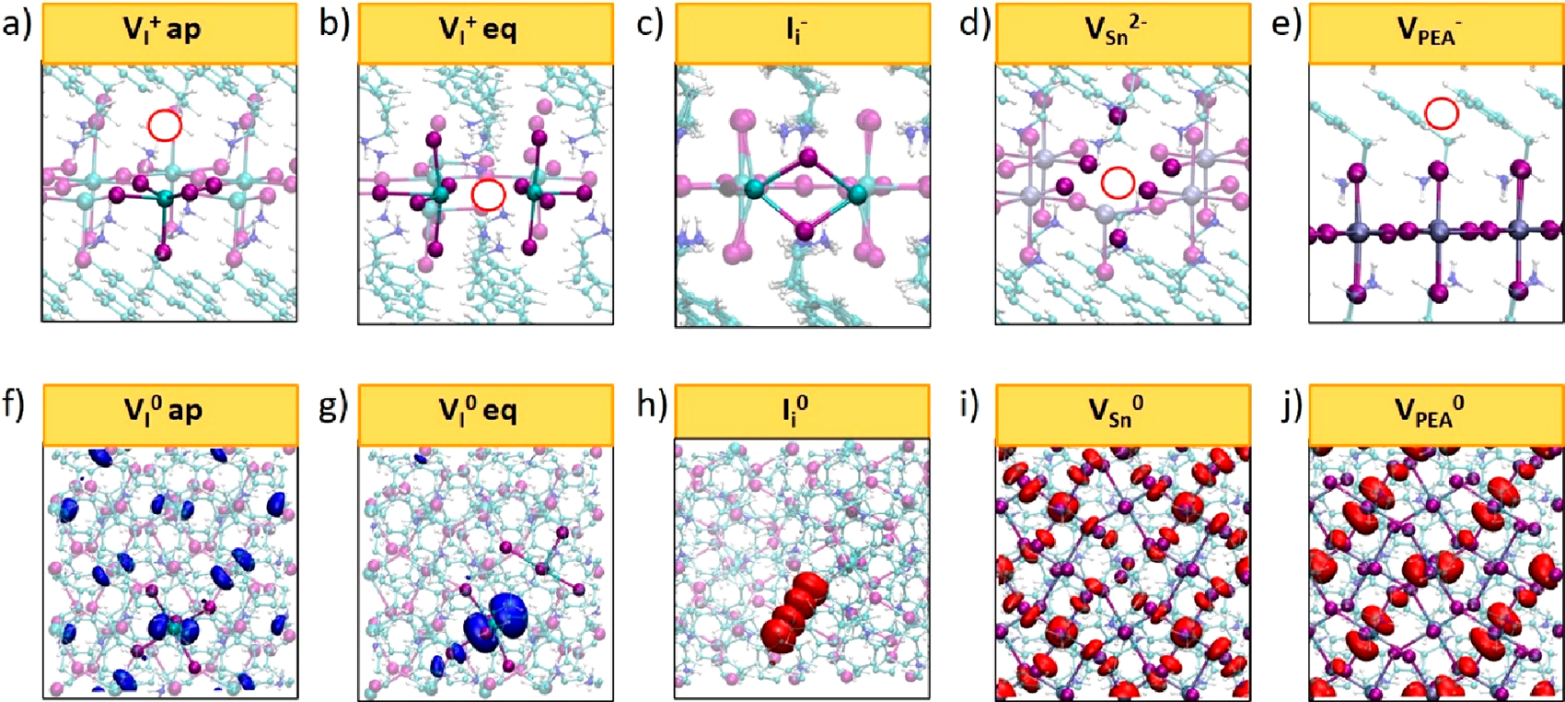
(a–e) Equilibrium structures of the most stable point defects in PEA_2_PbI_4_ and PEA_2_SnI_4_. KS plots of the trapped electron on the (f) apical and (g) equatorial iodine vacancy defects. KS plots of the trapped hole on (h) iodine interstitials. Delocalized hole for (i) tin vacancy and (j) PEA vacancy (orbital plot isovalue 0.03 au^–3/2^).

Iodine interstitials (I_i_; Figure 4c) are stable in their neutral charged state at the native Fermi level of PEA_2_PbI_4_, but the + and – charged states show larger fields of stability in the diagram in Figure 3a. Similar to interstitials in 3D MAPbI_3_,^30^ positive interstitial iodine I_i_^+^ is bonded with two lattice iodides to form a trimer at bond distances of ∼2.9 Å, i.e. I_3_^–^ molecules; in the neutral state, the interstitial is bonded to a lattice iodine at 3.1–3.2 Å to form I_2_^–^ radicals, and in the negative state, it is coordinated by two lead ions in the inorganic layer in a bridge configuration (see Figure S6 in the Supporting Information). Interestingly, the positive interstitial I_i_^+^ is also stable in the p region of the PEA_2_SnI_4_ diagram, in contrast to 3D MASnI_3_ (see Figure S4 in the Supporting Information), where only the negatively charged form of the defect I_i_^–^ is stable across the Fermi level range.^30^ In this case, the downshift of the VBM of PEA_2_SnI_4_ strongly destabilizes acceptor defects, such as I_i_^–^ and V_Sn_^2–^, compared to MASnI_3_ (see Figure S4 in the Supporting Information) by favoring the oxidized form of iodine interstitials. The deep (0/−) transitions located at 1.48 and 0.34 eV in PEA_2_PbI_4_ and PEA_2_SnI_4_, respectively, demonstrate that I_i_ may act as deep hole traps in the perovskites (see Figure 4h) and may increase nonradiative recombination in the materials.

Tin vacancies (V_Sn_; Figure 4d) are the most stable defects compensating iodine vacancies in PEA_2_SnI_4_. Similar to their lead counterpart, V_Sn_ are stable in the −2 charged state at the Fermi level. Although the neutral state of the defect is stable for a Fermi level close to the VB of the material, the KS plot of the holes does not show any localization on the defect (see Figure 4i), highlighting that V_Sn_ are shallow defects in PEA_2_SnI_4_, as is the case for 3D MASnI_3_. Differently from MASnI_3_, however, acceptor V_Sn_^2–^ are compensated by V_I_^+^, thus suggesting that PEA_2_SnI_4_ is not as heavily p doped as its 3D analogues.^30,31^

Lead and tin interstitials (Pb_i_/Sn_i_) show deep levels in the gap, but they have higher formation energies compared to halide defects and thus are likely to play a minor role in the defect chemistry. The cation vacancy V_PEA_ (Figure 4e) is not associated with significant rearrangement of the inorganic layer and does not introduce localized states in the band gaps of the materials (see Figure 4 j).

The analysis of the defect properties in these 2D perovskites shows deeper energy levels compared to 3D analogues due to the widening of the band gap in the former. Calculated DFEs, however, show that all modeled defects are remarkably unstable (DFE > 1 eV) at the native Fermi level. Under thermodynamic equilibrium conditions they will form in very low densities (∼10^2^ cm^–3^). As a comparison, calculated densities of ∼10^12^ and 10^16^ cm^–3^ were reported for the most stable defects in 3D-MAPbI_3_ and MASnI_3_, respectively.^30,32^

Such low defect densities in these 2D halide perovskites seem to contrast with the hypothesis of a defect origin of the BE. An out-of-equilibrium growth of the perovskites, however, may increase the density of defects in the film, particularly of halide vacancies. Notably, the growth kinetics of 2D perovskites are strongly influenced by the low diffusion constants of the PEA large cations in solution due to the associated large van der Waals radius. This is compatible with an increased density of halide vacancies, forming as energetically accessible compensating defects to PEA vacancies, and a decreased density of iodine interstitials. This hypothesis is in line with observations of Yin et al., showing that an excess of PEAI precursors in the solution leads to a quenching of the BE in PEA_2_PbI_4_ single crystals.^19^ Similarly, the BE intensity was shown to be affected by the precursor stoichiometry during thin-film formation.^33^

To support the halide vacancy hypothesis, we predicted all associated ionization levels and PL transitions from these defects for the four experimentally considered compounds. PL transitions have been evaluated by calculating the vertical emission associated with the recombination of the localized electron on the p orbital of the metal adjacent to the halide vacancy with a free hole in the VB: i.e., to V_X_^0^ + h^+^ = V_X_^+^.

Table 1 summarizes the calculated (+/0) ionization levels of the vacancies (in both equatorial and axial positions) and the predicted vertical PL emissions for all the examined PEA perovskites, with SOC corrections included. As reported in Table 1, all V_X_ defects show (+/0) transitions deep in the band gap, confirming that such defects can be activated upon photoexcitation. Comparing the calculated with the experimental PL emission energies, we find generally good agreement.

We find an excellent agreement between two calculated transitions at 1.75 and 0.82 eV and experimental values of 1.7 and 0.9 eV for PEA_2_SnI_4_. For PEA_2_PbI_4_, we observe a correspondence between one experimentally observed transition and a predicted value at 1.97 eV but fail to predict the pronounced band at 1.7 eV. DFT also predicts an emission by the equatorial vacancy at ∼1.3 eV in the near-infrared spectral region, similar to the case of PEA_2_SnI_4_.

In the case of PEA_2_PbBr_4_, the 1.75 eV emission matches well with the two calculated transitions of V_I_ but the higher energy band at 2.3 eV is better fitted by the STE emission. PEA_2_PbCl_4_ shows similarly good agreement with calculated emissions from V_I_ and its broad band peaking at 2 eV, but in this case the emission partially overlaps with the predicted STE emission, not allowing for a unique assignment.

As we summarize in the Jablonski diagram of Figure 5a, the generally good agreement between predicted and experimental emission energies supports the hypothesis that BEs are uniquely mediated by halide vacancy defects in PEA_2_SnI_4_ and PEA_2_PbI_4_, while additional and overlapping components due to emissions from STEs cannot be excluded in the case of PEA_2_PbBr_4_ and PEA_2_PbCl_4_ perovskites. Specifically, while in PEA_2_PbBr_4_ STE emission occurs at higher energies (2.19 eV) with respect to halide vacancy emission, it is not possible to disentangle the two phenomena based on their overlapping emission energies in the case of PEA_2_PbCl_4_, as we indicate in the stylized PL spectrum of Figure 5b.

**Figure 5:**
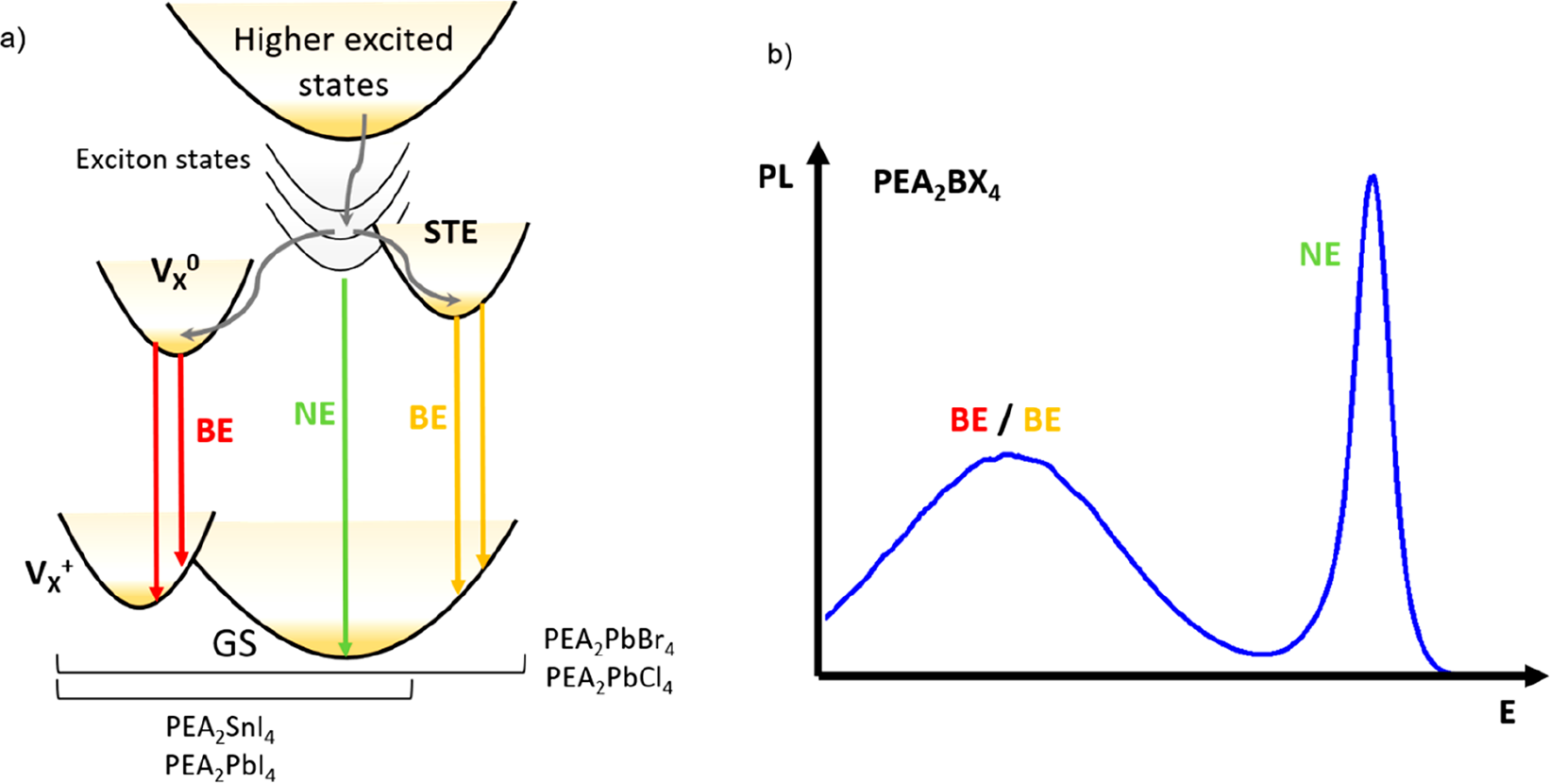
(a) Proposed Jablonski diagram for carrier dynamics and optical transitions in the PEA family of ⟨100⟩ 2D halide perovskites. Band to band transitions create the narrow emission (NE) of free excitons. Electron trapping at the V_X_ defect site and exciton self-trapping can result in broad emission, depending on the system; (b) Attribution of the emission bands to the transitions for the PEA_2_BX_4_ system.

The results of our analysis match with several experimental observations, by establishing a unified interpretation and new insights into the origin of BEs in 2D perovskites. The major role played by halide vacancies for the BE in PEA_2_PbI_4_ is in good agreement with previous works^13,14,19^ and the general observation that the presence of the BE depends on synthesis conditions. Halide-related defects furthermore are in line with the observed dependence on light bias,^13^ given the mobility of halide ions in perovskites.^34^ Also, our experiments on PEA_2_SnI_4_, reporting for the first time the PL peaks at ∼0.9 eV, match well with the calculated transition, by further supporting the V_I_ mechanism. We note that we studied this spectral region only after a transition at around 0.8 eV was predicted.

On the other hand, the existence of intrinsic processes resulting in BEs via STEs in PEA_2_PbBr_4_ and PEA_2_PbCl_4_ is in line with experiments performed by Zhang et al.^35^ and by Fu et al.,^36^ showing that in these phases the BE can be tuned mechanically by a pressure-induced stress of the lattice. This contrast is furthermore matched by a wider field of Br- and Cl-based 2D HaPs giving rise to BEs.^12^ The emission from PEA_2_PbCl_4_, present at room temperature, suggests the existence of a low barrier to STE formation compared to PEA_2_PbBr_4_, in agreement with the less shallow electron trapping energy found by our DFT calculations (see Table 1).

In summary, our analysis reveals that broad emission bands are ubiquitous in the ⟨100⟩ 2D metal halide perovskites based on phenylethylammonium spacers. They can show a complex behavior with more than one transition and nontrivial temperature dependence. Based on DFT calculations, we demonstrate that emission from halide vacancies is compatible with most of the experimentally observed optical features. Electron capture at a positive iodide vacancy with subsequent hole capture is the only radiative path explaining the BE in PEA_2_PbI_4_ and in PEA_2_SnI_4_. In the case of PEA_2_PbBr_4_ and PEA_2_PbCl_4_, emission from intrinsic STEs can introduce additional optical signatures. A STE-derived emission is expected to be more probable in PEA_2_PbCl_4_ than in PEA_2_PbBr_4_ due to the latter’s shallower ionization levels associated with the trapping of the hole/electron in the STE formation. Besides providing useful insights into the intrinsic and extrinsic mechanisms generating BEs in 2D perovskites, our analysis indicates that BEs can be modulated by controlling the crystallization process or by tuning PEA-halide stoichiometry in the precursor solution in order to control the kinetics of growth. In other words, optimization of synthesis protocols to reduce defect densities will facilitate the fabrication of bright narrow emitters, whereas efficient broad emitters, for example for white-light applications, require compositional engineering to create stable self-trapped excitons.

## Methods

### Thin Film Preparation

Preparations were carried out as detailed previously.^21^ All chemicals were used without further purification. Solutions and thin films were prepared inside a nitrogen-filled glovebox and deposited on quartz substrates.

### Absorption Spectroscopy

Absorption spectra were recorded with a Shimadzu 3600 UV–vis–NIR spectrometer.

### Photoluminescence Spectroscopy

The samples were mounted into a cryostat (Oxford HiRes microstat for the NIR measurements of PEA_2_SnI_4_; all others Oxford Optistat CF) and excited at 3.1 eV (400 nm) in case of the iodide-based compounds and at 4.6 eV (267 nm) for the bromide and chloride-based compounds using the second/third harmonic of a mode-locked Ti:sapphire laser (Mira 900, coherent) that emits at a repetition rate of 76 MHz. Steady-state spectra were recorded with a spectrally calibrated Hamamatsu EM-CCD camera in the visible and an Andor iDus 1.7 array detector in the near-infrared spectral region. The spectrometers were equipped with a 30 lines per millimeter grating. The excitation beam was spatially limited by an iris and focused with a lens of 150 mm focal length. The fluence was adjusted using gray filters and the spectra were taken in reflection with an incident angle of ∼60° with respect to the sample surface. Measurements were carried out under a helium atmosphere.

### Computational Details

DFT calculations have been carried out in the 2 × 2 × 1 supercells of the PEA perovskites by using the CP2K software package.^37^ The equilibrium structures of the self-trapped excitons and the defects have been found by relaxing ion positions with the PBE0 functional^27^ and by including DFT-D3 dispersions,^38^ keeping cell parameters fixed to the experimental values (PEA_2_SnI_4_*a* = 8.648 Å, *b* = 8.647 Å, *c* = 32.461 Å, α = 85.1°, β = 85.1°, γ = 89.5°;^39^ PEA_2_PbI_4_*a* = 8.739 Å, *b* = 8.740 Å, *c* = 32.995 Å, α = 84.6°, β = 84.6°, γ = 89.6°;^40^ PEA_2_PbBr_4_*a* = 11.615 Å, *b* = 11.628 Å, *c* = 17.575 Å, α = 99.5°, β = 105.7°, γ = 89.9°;^41^ PEA_2_PbCl_4_*a* = 11.115 Å, *b* = 11.205 Å, *c* = 17.591 Å, α = 99.1°, β = 104.5°, γ = 90.0°).^42^ In all cases DFT calculations have been performed at the Γ point by using the Goedecker–Teter–Hutter (GTH) norm-conserving pseudopotentials^43^ and double-ζ Gaussian basis sets.^44^ The auxiliary density matrix method^45^ has been used to accelerate hybrid functional calculations. DFEs and TILs of native point defects have been calculated by following the grand-canonical approach^23^ (see details in the Supporting Information).

Due to the large impact of spin–orbit coupling (SOC) on the CB energy level and on the ionization levels of CB-related defects in lead-based perovskites,^24,25^ the ionization levels of halide vacancies and of the self-trapped electron have been corrected for SOC effects by performing single-point PBE0-SOC calculations at the PBE0 relaxed geometries found with the CP2K code. As discussed in the Supporting Information, the use of the PBE0 functional together with the inclusion of SOC provides an accurate description of the electronic structure of 2D perovskites, predicting band gaps in good agreement with G_0_W_0_ calculations.

PBE0-SOC calculations have been performed at the Γ point by using the Quantum Espresso code.^46^ Norm-conserving full relativistic pseudopotentials were used (I 5s, 5p; Br 4s, 4p; Cl 3s, 3p; N and C 2s, 2p; H 1s; Pb 5s, 5p, 5d, 6s, 6p; Sn 4s, 4p, 4d, 5s, 5p shells explicitly included) with a cutoff on the wave functions of 40 Ry (80 Ry for the Fock grid) to reduce the computational effort.

Following the Franck–Condon principle, PL emissions from V_I_ and STE have been estimated by calculating the vertical emissions at the V_I_^0^ and STE equilibrium geometries, by using the PBE0 total energies of the systems (also see Figure S7). This approach has been shown to provide emission energies in good agreement with experiments.^47−49^ PL emission from halide vacancies is obtained by PL = ε(+/0) – (*E*^+^[V^0^] – *E*^+^[V^+^]), where ε(+/0) is the calculated TIL of the vacancy and the second term is the difference between the energy of the positive vacancy at the geometry of the neutral state *E*^+^[V^0^] and the energy of the positive vacancy at its equilibrium position *E*^+^[V^+^]. For STE the expression PL = *E*^T1^[STE] – *E*^S0^[STE] has been used, where *E*^T1^[STE] and *E*^S0^[STE] are the energies of the triplet and singlet states at the equilibrium geometry of the triplet. SOC corrections to PL energies have been estimated by rigidly subtracting from the PBE0 PL emission the energy shifts of the (+/0) and (0/−) transitions obtained by PBE0-SOC calculations for V_I_ and the trapped electrons in STE, respectively. It is worth mentioning that the accuracy in the PL prediction is largely determined by the accuracy in the description of the electronic structure of the perovskites. In this regard, the high-quality results provided by our approach, combining the use of hybrid functionals and SOC corrections, are expected to reproduce experimental results at low temperature with a good accuracy.

## Supporting Information Available

PL and absorption spectrum of PEA_2_PbCl_4_, power-dependent intensity of the near-infrared emission of PEA_2_SnI_4_, additional information on the computational approach and the electronic structure of PEA_2_PbI_4_ and PEA_2_SnI_4_, comparison of the DFEs and TILs with MASnI_3_ and MAPbI_3_, comparison of calculated transition energies with and without SOC, unit cell parameters and atomic coordinates for optimized structures, calculated values of high- and low-frequency dielectric constants, and illustration of the calculated states’ energy and transitions (PDF)
