# Supplementary material for: The Origin of Broad Emission in ⟨100⟩ Two-Dimensional Perovskites: Extrinsic vs Intrinsic Processes
Source: ACS Energy Lett. 2022 Oct 31;7(12):4232–41. doi: 10.1021/acsenergylett.2c02123 (PMC9745793; doi:10.1021/acsenergylett.2c02123)
Supplement: Supplementary file 1 — nz2c02123_si_001.pdf [file nz2c02123_si_001.pdf]

Supporting Information for

The Origin of Broad Emission in <100> Two-Dimensional Perovskites: Extrinsic vs. Intrinsic Processes

Simon Kahmann,[1,2,#,*] Daniele Meggiolaro,[3,#,*] Luca Gregori,[4] Eelco K. Tekelenburg,[1] Matteo Pitaro,[1] Samuel D. Stranks,[2,5] Filippo De Angelis,[3,4,6] and Maria A. Loi[1]

[1] *Photophysics and OptoElectronics Group, Zernike Institute for Advanced Materials, University of Groningen, Nijenborgh 4, 9747 AG Groningen, The Netherlands*

[2] *Cavendish Laboratory, University of Cambridge, JJ Thomson Avenue CB3 0HE, Cambridge, UK*

[3] *Computational Laboratory for Hybrid/Organic Photovoltaics (CLHYO), Istituto CNR di Scienze e Tecnologie Chimiche (SCITEC-CNR), Via Elce di Sotto 8, 06123, Perugia, Italy.*

[4] *Department of Chemistry, Biology and Biotechnology, University of Perugia, Via Elce di Sotto 8, 06123, Perugia, Italy.*

[5] *Department of Chemical Engineering and Biotechnology, University of Cambridge, Philippa Fawcett Drive CB3 0AS, Cambridge, UK*

[6] *Department of Natural Sciences & Mathematics, College of Sciences & Human Studies, Prince Mohammad Bin Fahd University, Saudi Arabia.*

# These authors contributed equally.

* Corresponding authors

Email: sk2133@cam.ac.uk, daniele.meggiolaro@cnr.it

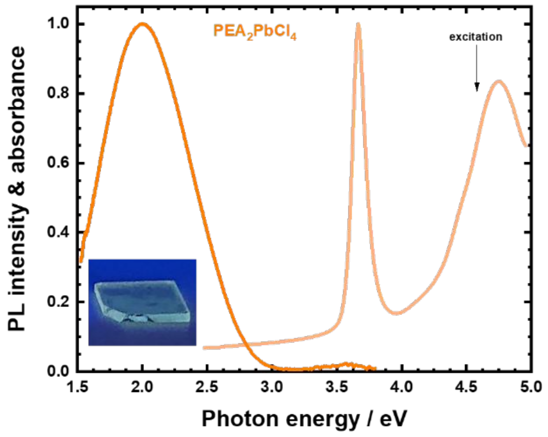

Figure S1: Absorbance and photoluminescence spectrum of PEA_2PbCl_4 (excited at 4.6 eV) at room temperature.

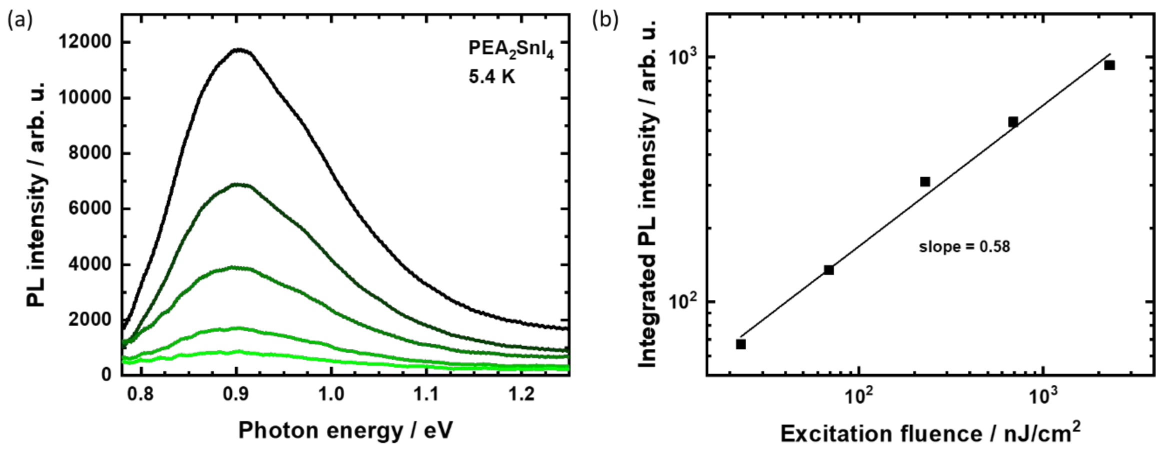

Figure S2: Power-dependent PL of the BE of PEA_2SnI_4 at 5.4 K. Steady state spectra (a) and integrated intensity on a double-logarithmic plot indicating the sub-linear dependence.

DFT simulations

Electronic structure of $(PEA)_2SnI_4$ and $(PEA)_2PbI_4$

The impact of the level of theory on the electronic properties of 2D perovskites is illustrated in the following for the PEA_2SnI_4 and PEA_2PbI_4 phases. Calculations have been carried out on the relaxed structures of the phases obtained by using the PBE functional (DFT-D3 dispersions included) and by fixing cell parameters to the experimental values.

At the PBE level of theory, spin-orbit coupling (SOC) included, the PEA_2SnI_4 and PEA_2PbI_4 phases show direct band gaps at the Γ point in the Brillouin zone (BZ) of 1.32 eV and 0.99 eV, respectively, see Figure S3 and Table S1. Similar to the 3D bulk phase, the top of the valence band (VBM) is associated to I-p orbitals, while the conduction band minimum (CBM) is mainly associated with the p-states of the metal. As expected, the calculated band gaps at the PBE-SOC level are underestimated, as demonstrated by the higher optical band gaps measured in absorption experiments.

In order to provide a more accurate prediction of the optoelectronic properties of the phases, the band gaps of the PEA_2PbI_4 and PEA_2SnI_4 phases have been calculated by the G_0W_0 method, by using the Yambo code.[1]

In Table S1 the calculated band gaps at the G_0W_0-SOC level are reported. A renormalization of the band gaps to values of 1.88 and 2.51 eV is reported for PEA_2SnI_4 and PEA_2PbI_4 , respectively. The calculated band gaps are in good agreement with experimental works reporting values of 2.08 and 2.61 eV for PEA_2SnI_4 and PEA_2PbI_4, respectively.[2]

The accuracy of hybrid functionals in the description of the electronic structure of the phases has been checked by recalculating the band gaps at the PBE0-SOC level, see Table S1. A good agreement with G_0W_0 calculations is found for lead, while for tin perovskite the band gap is slightly overestimated by the PBE0 functional. This analysis shows that PBE0 functional (SOC included) provides an accurate description of the electronic properties of these systems. Notably, small deviations of the calculated PBE0-SOC band gaps of PEA_2SnI_4 and PEA_2PbI_4 can be noticed between Table S1 and Table 1 of the main text. These variations are due to the slightly different structures of the phases obtained at the PBE and PBE0 levels of theory (band gaps reported in Table 1 of the main text have been calculated at the PBE0 relaxed structures obtained by the CP2K code).

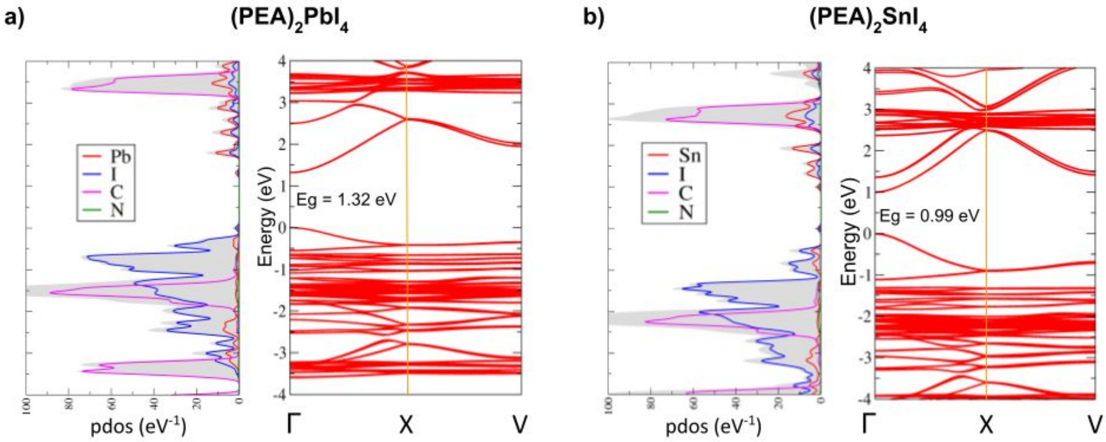

Figure S3. Projected density of states (PDOS) and electronic band structure of the $(PEA)_2PbI_4$ and $(PEA)_2SnI_4$ phases calculated at the PBE-SOC level of theory.

Table S1. Electronic band gaps of 2D perovskites calculated at different levels of theory.

System	PBE-SOC (eV)	PBE0-SOC (eV)	G_0W_0-SOC (eV)	Experiments (eV)
$(PEA)_2SnI_4$	0.99	2.12	1.88	2.08[2]
$(PEA)_2PbI_4$	1.32	2.57	2.51	2.61[2]

Defect calculations

Defect formation energies (DFE) and thermodynamic ionization levels (TIL) of defects were calculated by using the following relations[3]

$$DFE\ [X^q] = E[X^q] - E[perf] - \sum_i n_i\mu_i + q(\varepsilon_{VB} + \varepsilon_F) + E^q_{corr} \quad (1)$$

$$\varepsilon(q/q') = \frac{E[X^q] - E[X^{q'}]}{q' - q} + \frac{E^q_{corr} - E^{q'}_{corr}}{q' - q} - \varepsilon_{VB} \quad (2)$$

where $E[X^q]$ is the energy of the supercell with defect X in the charge state q; $E(perf)$ is the energy of the perfect (non-defective) supercell; n and μ are, respectively, the number and the chemical potentials of the species added or subtracted to the non-defective system; ε_{VB} and ε_F are the valence band energy and the Fermi level; $E^q{}_{corr}$ is the correction term due to the charge. Charge corrections have been applied by following the Makov-Payne scheme by using the ionic dielectric constant of the phases calculated at the PBE level by following the approach of Umari et al[4] (see Table S4).

The chemical potentials of the elements in the calculation of PEA_2SnI_4 and PEA_2PbI_4 DFEs have been set by imposing the thermodynamic stability of the 2D perovskites and the equilibrium between the 2D perovskites and the relative metal precursors (SnI_2 and PbI_2)

$$2\mu(PEA) + \mu(Pb/Sn) + 4\mu(I) = \mu(PEA_2(Pb/Sn)I_4) \quad (3)$$

$$\mu(Pb) + 2\mu(I) = \mu(PbI_2) \quad (4)$$

$$\mu(Sn) + 2\mu(I) = \mu(SnI_2) \quad (5)$$

I-rich conditions have been simulated by setting the chemical potential of iodine to the value in solid I_2 ($\mu(I) = ½ \mu(I_2)$), while I-poor conditions by setting the chemical potentials of lead / tin to the values in the respective metal bulks. The chemical potentials of other species have been calculated by considering equilibriums 3-5. For I-medium conditions the average values of the chemical potentials between I-rich and I-poor conditions have been used.A comparison of the calculated DFEs (I-medium conditions) and TILs in the 2D perovskites and in the 3D analogues is reported in Figures S4 and S5.

The electronic structure analysis highlights that quantum confinement into two dimensions increases the band gap energy through a down/up-shift of the VBM/CBM for both tin and lead perovskites compared to their 3D counterparts (see Figure S4). At the PBE0 level of

theory, spin-orbit corrections (SOC) included, a down-shift of 0.42 eV and an upshift of 0.45 eV are observed for the VBM and CBM of PEA_2PbI_4 with respect to 3D $MAPbI_3$ (see Figure S4 and Figure S5). Shifts of 0.59 and 0.25 eV of the VBM and CBM are observed in the case of PEA_2SnI_4. These shifts and the associated band gaps opening leads to a widening of the stability field of defects and to a deepening of the VB and CB-related ionization levels compared with 3D analogues. As an example, (+/0) levels of V_I, which are shallow in $MAPbI_3$, become deep in PEA_2PbI_4 ; (0/-) transitions associated to I_i, which are shallow in $MASnI_3$, become deep in PEA_2SnI_4.

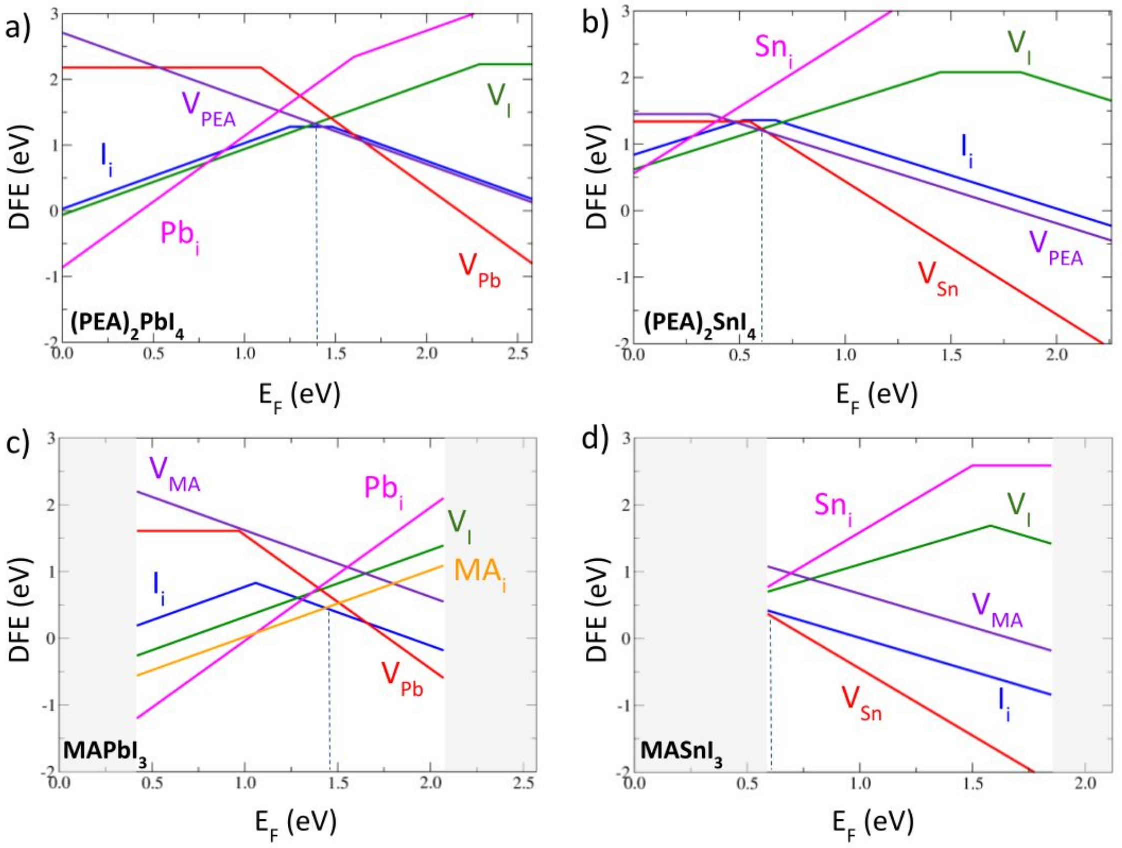

Figure S4. a-b) Defect formation energies of 2D and c-d) 3D perovskites calculated in I-medium condition at the PBE0 level. The band gaps and VB / CB alignments are those calculated at the PBE0-SOC level of theory.

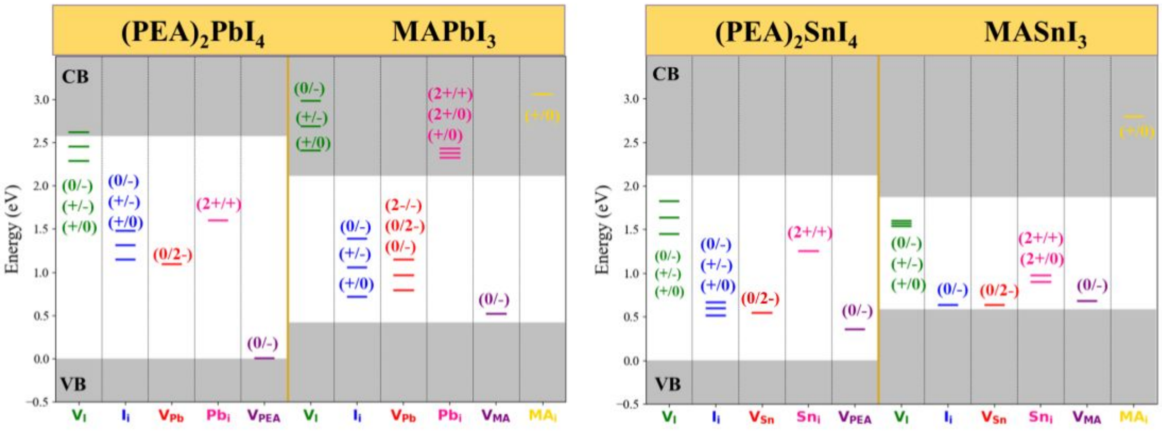

Figure S5. Thermodynamic ionization levels of main defects in 2D and 3D lead and tin perovskites, calculated at the PBE0 level of theory. The band gaps and VB / CB alignments are those calculated at the PBE0-SOC level of theory

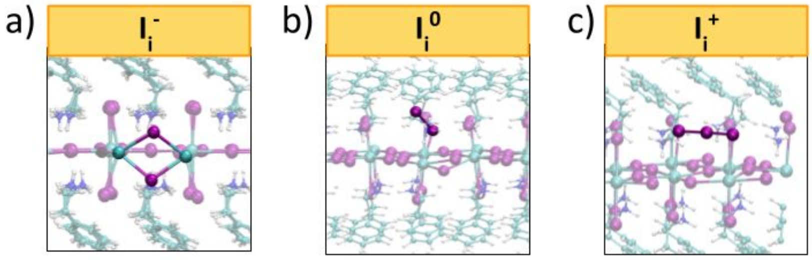

Figure S6. Equilibrium structures of the iodine interstitial in the a) negative; b) neutral and c) positive charged forms.

Table S2. Predicted band gaps; (0/-) and (+/0) transitions of STEs; (+/0) transitions of halogen vacancies (equatorial = eq; apical = ap); calculated PL emission energies of the STE and V_X. A comparison of the values calculated at the PBE0 level of theory with and without SOC (in parenthesis) is provided.

**Phase**	**TIL / eV**	**PL emission Theory / eV**
PEA_2SnI_4 E_g = 2.26 eV (2.42 eV)		
STE	-	-
V_I eq	(+/0) 1.42 (1.45)	0.82 (0.85)
V_I ap	(+/0) 1.84 (1.99)	1.75 (1.90)
PEA_2PbI_4 E_g = 2.58 eV (3.33 eV)		
STE	-	-
V_I eq	(+/0) 1.94 (2.29)	1.27 (1.62)
V_I ap	(+/0) 2.07 (2.86)	1.97 (2.76)
PEA_2PbBr_4 E_g = 3.26 eV (4.13 eV)		
STE	(0/-) 3.48 (4.01) (+/0) (0.05)	2.19 (2.72)
V_{Br} eq	(+/0) 2.56 (2.79)	1.78 (2.02)
V_{Br} ap	(+/0) 2.93 (3.30)	1.73 (2.11)
PEA_2PbCl_4 E_g = 3.91 eV (4.62 eV)		
STE	(0/-) 3.88 (4.21) (+/0) (0.12)	1.89 (2.23)
V_{Cl} eq	(+/0) 2.89 (3.02)	1.97 (2.09)
V_{Cl} ap	(+/0) 3.29 (3.52)	1.85 (2.08)

Table S3. Optimized cell parameters and band gaps at different levels of theory (PBE and PBE+SOC) with the inclusion of DFT-D3 dispersions. DFT relaxations have been performed by using the CP2K and Quantum Espresso codes (small variations in the optimized cell parameters are ascribed to the different used pseudopotentials for Pb, i.e. 4 valence electrons CP2k vs 14 valence electrons QE). Variations of the DFE and (+/0) TIL energy of V_I eq in fully relaxed supercells with respect to values calculated with cell parameters fixed at the experimental values.

	a, b, c, α, β, γ	E_{gap} (eV)
Experimental cell	a=8.739 Å, b=8.740 Å, c=32.995 Å, α=84.6°, β=84.6°, γ=89.6°	2.13
Optimized cell (PBE-D3, CP2K)	a=8.650 Å, b=8.633 Å, c=32.260 Å, α=85.6°, β=85.8°, γ=89.2°	2.12
Optimized cell (PBE-D3, QE)	a=8.694 Å, b=8.688 Å, c=32.945 Å, α=85.5°, β=85.5°, γ=89.3°	2.13
Optimized cell (PBE-SOC-D3, QE)	a=8.694 Å, b= 8.688 Å, c=32.945 Å, α=85.5°, β=85.5, γ=89.3°	1.32
Defect	ΔDFE (eV)	Δ(+/0) TIL (eV)
V_I^+ eq (PBE-D3, CP2K)	+0.02	-0.10
V_I^0 eq (PBE-D3, CP2K)	-0.08	

Table S4. Calculated high frequency (ε_∞) and static (ε_0) dielectric constants for the studied 2D phases at the PBE level. In the last column the Makov-Payne (MP) corrections to charged defect energies (charge q) in the respective supercells are also reported.

**System**	ε_∞	ε_0	MP correction (eV)
**PEA_2SnI_4**	ε_{xx} = 4.3<br>ε_{yy} = 4.3<br>ε_{zz} = 3.1	ε_{xx} = 10.3<br>ε_{yy} = 10.2<br>ε_{zz} = 4.2	(q=1) +0.05<br>(q=2) +0.22
**PEA_2PbI_4**	ε_{xx} = 3.6<br>ε_{yy} = 3.6<br>ε_{zz} = 3.0	ε_{xx} = 7.1<br>ε_{yy} = 7.2<br>ε_{zz} = 4.0	(q=1) +0.10<br>(q=2) +0.40
**PEA_2PbBr_4**	ε_{xx} = 3.2<br>ε_{yy} = 3.2<br>ε_{zz} = 2.7	ε_{xx} = 6.5<br>ε_{yy} = 6.7<br>ε_{zz} = 3.8	(q=1) +0.10
**PEA_2PbCl_4**	ε_{xx} = 3.0<br>ε_{yy} = 3.0<br>ε_{zz} = 2.7	ε_{xx} = 7.4<br>ε_{yy} = 7.3<br>ε_{zz} = 3.9	(q=1) +0.16

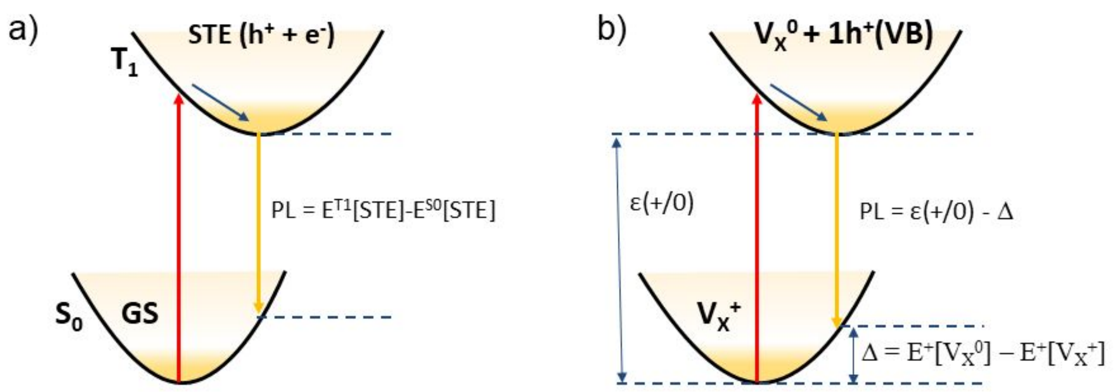

Figure S7. Diagrams representing the simulated PL emission from a) STE and b) halide vacancy. E^{T1}[STE] and E^{S0}[STE] represent the energies of the triplet and singlet states, respectively, calculated at the equilibrium geometry of the STE; $E^{+}[V_X{}^{0}]$ and $E^{+}[V_X{}^{+}]$ represent the energies of the positive vacancy at the equilibrium geometries of the neutral and positive halide vacancy, respectively.

Table S5. Optimized structures of the positive / neutral equatorial and apical iodine vacancies in PEA_2PbI_4 and the self-trapped exciton in PEA_2PbCl_4, as calculated in the 2x2x1 supercells at the PBE0-D3 level of theory and by fixing cell parameters to the experimental values. Calculations have been carried out by using the CP2K code (version 6.1). Ion positions and supercell vector parameters (a, b, c) are in Å. Hydrogens have been removed for brevity.

PEA_2PbI_4	PEA_2PbCl_4
a = 17.478 0.000 0.000<br>b = 0.110 17.480 0.000<br>c = 3.071 3.057 32.709	a = 22.230 0.000 0.000<br>b = 0.000 22.410 0.000<br>c = -4.413 -2.803 16.796

(V_I^+eq)

Pb	-0.0015650905	0.0042996031	0.0221747176
Pb	4.3964522389	4.3678529535	0.0226066146
Pb	5.9004579328	1.4747229046	16.3873210323
Pb	1.5919637320	5.8907041449	16.3918029626
Pb	0.0532591421	8.7440304950	0.0226679387
Pb	4.4519906384	13.1078591906	0.0228052575
Pb	5.9654941519	10.2701296793	16.3852808648
Pb	1.6637548675	14.6412739829	16.3849582540
Pb	8.7378960287	0.0045203506	0.0218487530
Pb	13.1358211772	4.3683315766	0.0231553205
Pb	14.6751338699	1.4819959576	16.3781903244
Pb	10.2308742865	5.8489124833	16.3648452663
Pb	8.7916913584	8.7432539311	0.0221027233
Pb	13.1906138510	13.1076631664	0.0219873211
Pb	14.7508767421	10.3671460636	16.4195522589
Pb	10.3517953961	14.6241429948	16.3760922916
I	6.0193075775	7.1324886425	0.0448265602
I	4.1702317777	4.5047961225	3.2412978717
I	7.7037554085	7.2918045787	29.5115143175
I	2.8485937264	2.9128103933	29.5069027802
I	9.0236477411	8.8903765970	3.2494227423
I	8.7827438618	8.6838350989	16.3907681775
I	3.1481093257	3.1031796878	16.3814832849
I	10.5079766617	5.7549307463	19.5518331329
I	1.5007999847	6.1319786758	13.1802737885
I	5.7716339944	1.3195742203	13.1898020572
I	6.1066756624	10.4713054977	19.5886539541
I	7.1763565891	2.7879762358	0.0616542247
I	4.3757627350	7.4631533353	16.4244670092
I	7.6131992471	4.3331574502	16.3677324584
I	1.6228272427	5.9615597578	0.0135783027
I	2.7761990849	1.6053310386	-0.0328021758
I	6.0758574255	15.8720070203	0.0461004282
I	4.2253896092	13.2442137982	3.2417135514
I	7.7594823581	16.0311961526	29.5120601884
I	2.9034049698	11.6523614709	29.5073700783
I	9.0797620791	17.6308237064	3.2473648225
I	8.8099398100	17.4256263068	16.4154336552
I	3.1924443937	11.8394450982	16.3773071606
I	10.4848534429	14.3971859608	19.5782505826
I	1.4957294034	14.8715577242	13.1918748034
I	5.8165309677	10.0385938152	13.1731003824
I	6.1802105701	19.2345127260	19.5803931832
I	7.2311571570	11.5269470063	0.0624675010
I	4.4155173655	16.2194494808	16.4271668100
I	7.5614584552	13.0555302371	16.3414039182
I	1.6792274072	14.7025394026	0.0126236296
I	2.8307968364	10.3456404381	-0.0326896915
I	14.7584736050	7.1333262808	0.0469860004
I	12.9095581401	4.5045215654	3.2425165952
I	16.4425189432	7.2917302133	29.5124434571
I	11.5880551312	2.9127802950	29.5059892184
I	17.7630408969	8.8904258537	3.2483965422
I	17.4525112620	8.6736910279	16.4116923751
I	11.9217823047	3.1517395888	16.3533726454
I	19.1484018129	5.6573602155	19.5910843586
I	10.2740866938	6.1420265533	13.1775060064
I	14.5135252279	1.3207380713	13.1799454136
I	14.8214899776	10.4565681701	19.6059245148
I	15.9151904890	2.7879160290	0.0620112138
I	16.1831769259	4.3722080379	16.3300290546
I	10.3617153593	5.9612112597	0.0138328185
I	11.5159125199	1.6055691665	-0.0322217639
I	14.8142662507	15.8716857643	0.0454981122
I	12.9634334272	13.2450840067	3.2416423621
I	16.4981333524	16.0314791618	29.5115852169
I	11.6429016744	11.6515672802	29.5070925034
I	17.8185050286	17.6315064941	3.2486877206
I	17.5575478901	17.4242685449	16.4102581992
I	11.8981635945	11.7762716718	16.3494595551
I	19.2120294464	14.3970392338	19.5790918698
I	10.2469999261	14.8752968811	13.1742947448
I	14.4668756255	9.9452107024	13.2446209513
I	14.9205202604	19.1304301003	19.6097740770
I	15.9701891699	11.5276981246	0.0621286291
I	13.1658871053	16.2066504380	16.4198103669
I	16.2778825367	12.9847403386	16.3260058147
I	10.4172590373	14.7018507998	0.0133560276
I	11.5688992573	10.3455518486	-0.0335218352
N	11.2193168443	8.0920394735	30.0728769384
N	0.6525131385	3.7133465924	2.7231148303
N	8.1089136926	3.7218340128	30.0702942025
N	3.7567846724	8.0741404225	2.6415306010
N	9.6200907841	2.2275758662	19.0228792489
N	2.2528636946	9.6498163937	13.7104749632
N	5.2647736705	5.2438320273	19.0868289015
N	6.6815075312	6.5693648051	13.7069169859
N	11.2744163096	16.8319187682	30.0730787572
N	0.7076250565	12.4534574702	2.7226366727
N	8.1638049515	12.4620060082	30.0703417557
N	3.8116634377	16.8136633815	2.6414771387
N	9.6692076354	10.9007665270	19.0938524724
N	2.3068338358	18.3928111087	13.7092975751
N	5.3277122635	13.9777767017	19.0869075667
N	6.6597599834	15.2968507924	13.6749311689
N	19.9583626542	8.0920168925	30.0729690747
N	9.3915370499	3.7132732751	2.7229723520
N	16.8478791122	3.7218488914	30.0704733623

(V_I^0eq)

Pb	-0.0022030331	0.0046949629	0.0220539563
Pb	4.3967595744	4.3694055387	0.0219308679
Pb	5.9337999625	1.6347974989	16.3585767367
Pb	1.5937061846	5.8917402263	16.3461759859
Pb	0.0523974382	8.7452371526	0.0215172884
Pb	4.4524852494	13.1086074986	0.0240048680
Pb	5.9991168283	10.2627315908	16.3537732758
Pb	1.6396811101	14.6083932701	16.3534369062
Pb	8.7365442511	0.0052376983	0.0215830989
Pb	13.1366985434	4.3689549164	0.0234640567
Pb	14.6233364087	1.5477410841	16.3417552102
Pb	10.7633049749	6.1815099868	16.3139339193
Pb	8.7918075389	8.7447947354	0.0220261480
Pb	13.1908048647	13.1091164206	0.0220397646
Pb	14.6286129456	10.2345559458	16.4141168395
Pb	10.3385975403	14.6682440510	16.3666497257
I	6.0173544619	7.1356134738	0.0465213557
I	4.1684162259	4.5103100717	3.2383882180
I	7.7058158610	7.2901301388	29.5148023637
I	2.8469148239	2.9104310956	29.5111680954
I	9.0245195069	8.8937318208	3.2441736231
I	8.7835887666	8.7455174627	16.3835132117
I	3.0853572804	3.0971787482	16.3146183362
I	10.5631894390	5.8130085819	19.5806061735
I	1.4852545431	6.1334344743	13.1243030433
I	5.7432792439	1.3070162496	13.1343927907
I	6.1101228535	10.4802702503	19.5771767173
I	7.1770734253	2.7901425734	0.0612801704
I	4.3534830901	7.4927924060	16.3869003127
I	7.6099952384	4.1743880462	16.3090441360
I	1.6220086979	5.9628124219	0.0133129634
I	2.7754436980	1.6073650596	-0.0338089473
I	6.0725440384	15.8752247555	0.0476606756
I	4.2235289721	13.2483234183	3.2412176535
I	7.7602727275	16.0296039569	29.5168516090
I	2.9013161071	11.6507448266	29.5094473992
I	9.0778217745	17.6311612963	3.2422213291
I	8.7930855040	17.4216934934	16.4277114660
I	3.2041664923	11.8261933640	16.3473559163
I	10.4938916266	14.4051436318	19.5767112182
I	1.4578521423	14.8117667697	13.1302400530
I	5.8131846974	10.0368158347	13.1350931072
I	6.1705169420	19.2368240167	19.5931017681
I	7.2324022570	11.5290529925	0.0617715567
I	4.2818902921	16.1999409699	16.3756905708
I	7.5673773460	13.0626973810	16.3252380641
I	1.6785816753	14.7024461061	0.0116807726
I	2.8303200992	10.3472991863	-0.0337959614
I	14.7564841851	7.1359149737	0.0475945625
I	12.9079419203	4.5088298510	3.2403649799
I	16.4446402802	7.2899935297	29.5157606586
I	11.5856358597	2.9114324821	29.5095406876
I	17.7616332671	8.8927397170	3.2433809854
I	17.4125709058	8.6465890083	16.3756283918
I	11.9268644893	3.1575447127	16.3175595442
I	19.1600055304	5.6533605299	19.5570528333
I	10.2721116529	6.2058670969	13.0672102907
I	14.4966607955	1.3191698468	13.1249378017
I	14.8313972284	10.4691305586	19.6203908886
I	15.9164005113	2.7890662650	0.0617519615
I	16.2011298934	4.3760505734	16.3058972796
I	10.3633705255	5.9634107086	0.0107804908
I	11.5144282912	1.6079202128	-0.0344901416
I	14.8115135719	15.8751307438	0.0472240202
I	12.9631806309	13.2502186892	3.2384871845
I	16.4995395936	16.0287689127	29.5144833552
I	11.6409794151	11.6507838793	29.5105884541
I	17.8174684101	17.6336536583	3.2451072268
I	17.4985864589	17.3556041933	16.3937285049
I	11.8975686700	11.8193422586	16.3281098902
I	19.2055150696	14.3686475737	19.5683544252
I	10.2279058695	14.8904245366	13.1459909633
I	14.4624115993	9.9602762083	13.2073421899
I	14.9081463607	19.2430752718	19.5713774946
I	15.9712237965	11.5302124434	0.0617799964
I	13.1506615338	16.2329110270	16.3867688888
I	16.2378969550	12.8173297796	16.2601195183
I	10.4162591570	14.7024204520	0.0130324759
I	11.5694904140	10.3472115328	-0.0337076545
N	11.2194402426	8.0922105038	30.0726728247
N	0.6528099843	3.7137242236	2.7230918498
N	8.1092397983	3.7222454620	30.0706456849
N	3.7548004444	8.0743655260	2.6419820534
N	9.6123598551	2.3288296640	18.9897887614
N	2.2533347945	9.6461382005	13.7141366132
N	5.2666946557	5.2280592077	18.9888991851
N	6.7012378588	6.5531959048	13.7226043066
N	11.2742047834	16.8323584422	30.0727622052
N	0.7079644962	12.4539066116	2.7223306464
N	8.1641277422	12.4623364304	30.0707376718
N	3.8097017364	16.8138311404	2.6417236808
N	9.6718886167	10.8989084774	19.0822667642
N	2.3070861712	18.3004680592	13.7143527812
N	5.3113315419	13.9740907265	18.9951945658
N	6.6571563624	15.3073242691	13.7145449273
N	19.9584530046	8.0920268213	30.0725579722
N	9.3922432217	3.7139551403	2.7217992290
N	16.8482771767	3.7222364782	30.0705961993

(STE)

Pb	11.6756755509	18.1288737938	8.5661029880
Pb	17.2584800632	18.4125920275	8.3707037961
Pb	11.6912665075	12.4901811122	8.3821975686
Pb	17.2361451936	12.7221760682	8.1797741132
Pb	11.7057801481	6.9386790315	8.5935788499
Pb	17.2658788854	7.1581582505	8.3800473648
Pb	11.6599448850	1.2548683053	8.4335117078
Pb	17.1391080243	1.4651004372	8.1748965795
Pb	0.5767980381	18.1070056779	8.6161619232
Pb	6.1046475974	18.4146983158	8.3915540818
Pb	0.5746173544	12.4421520098	8.3772470282
Pb	6.0986126074	12.7545275277	8.1854934367
Pb	0.5987680474	6.8699810033	8.5722494490
Pb	6.1420904014	7.1803621242	8.3873914094
Pb	0.5811565585	1.1759952713	8.2477171752
Pb	6.2592857232	1.5636105450	8.1827204512
Cl	12.4337130263	21.0232264907	8.3721154927
Cl	16.3725920999	9.9874283834	8.3705109176
Cl	8.8588100612	11.9351671346	8.6400132284
Cl	14.4134806296	13.4277260365	8.4598546851
Cl	14.4669292827	17.4924443848	8.2800886125
Cl	8.9676604791	19.0833496781	8.0847339700
Cl	11.6955691403	12.5262466620	11.2265948362
Cl	17.2307917152	18.3496180339	5.5043814724
Cl	11.6360200690	12.6200452329	5.4715516439
Cl	17.3380141867	18.3142939660	11.2660088488
Cl	10.9539293261	15.3377580661	8.3811936365
Cl	17.9468118833	15.5954432110	8.3924420864
Cl	11.5058560400	18.3017933811	11.3216538826
Cl	17.3954079447	12.5534565669	5.4164256938
Cl	11.7893540056	17.8965522153	5.5388415550
Cl	17.1089952909	12.9827687929	11.2361822547
Cl	12.4864916067	9.6430086883	8.3553186096
Cl	16.1590767910	-1.2545324760	8.3058926974
Cl	9.0946190678	0.5627360158	8.6824380461
Cl	14.2116851326	2.1537039086	8.4776262446
Cl	14.4970035098	6.2229038869	8.2973235360
Cl	9.0039667800	7.7872650207	8.1496413464
Cl	11.7083290966	1.3064459223	11.1782832472
Cl	17.2290001416	7.1444564101	5.5200376398
Cl	11.5274982200	1.2902628927	5.7014021687
Cl	17.3037565838	7.0668056084	11.2955304351
Cl	10.9542205361	3.9470757096	8.3844603596
Cl	17.9703230043	4.3575485559	8.3802837863
Cl	11.5304818055	7.1167925519	11.3409678074
Cl	17.3825355959	1.3282236472	5.4079268263
Cl	11.8109571682	6.6759365196	5.5368786077
Cl	17.0435595488	1.7666494095	11.2373871185
Cl	1.4641584304	20.7474130400	8.3598839965
Cl	5.3417454449	9.9991016077	8.3801712433
Cl	-2.2857402449	11.8400644536	8.6642952735
Cl	3.3004072650	13.4207214536	8.4699781401
Cl	3.4326172877	17.4122640437	8.2971193511
Cl	-2.1535878191	18.8854917003	8.2048978369
Cl	0.5904419943	12.4787935087	11.2307639067
Cl	6.1199986810	18.3456796035	5.5244158466
Cl	0.5323637351	12.5947780548	5.4493310601
Cl	6.2047672603	18.2699163746	11.3080489052
Cl	-0.2339468782	15.2032961690	8.3673473439
Cl	6.9362113730	15.5987296591	8.4268910114
Cl	0.3545398819	18.3536834089	11.3673492605
Cl	6.2873722278	12.5688689878	5.4174882116
Cl	0.6784249518	17.8831993918	5.5382191351
Cl	6.0026960422	12.9657272189	11.2369916376
Cl	1.4561098308	9.6601985543	8.3572589223
Cl	5.3764572532	-1.1986440154	8.4007075383
Cl	-2.7408925800	0.3475807354	8.7354958997
Cl	3.7569515819	2.3962230379	8.4001762823
Cl	3.4379439844	6.2619466698	8.2914509806
Cl	-2.1502153968	7.7261864724	8.1547824241
Cl	0.5500711507	1.2318514389	11.2239999605
Cl	6.1473561032	7.1394600377	5.5375637488
Cl	0.5639082024	1.3637468737	5.2595162026
Cl	6.2068111749	7.0665819477	11.3181021583
Cl	-0.1840802527	4.1004099590	8.3161076503
Cl	7.0586208207	4.4188668823	8.4264425608
Cl	0.3927154803	7.0734089183	11.3496037428
Cl	6.4498500156	1.2331881718	5.4392635142
Cl	0.6992327292	6.6702955001	5.5329386911
Cl	6.0721581994	1.7724992978	11.2954313827
N	8.9188284390	14.3443773009	10.8458444395
N	20.0067377159	16.4771408901	5.9488082408
N	15.4246972199	15.6706236094	10.7667166097
N	13.5344548468	15.2438068352	5.9477577452
N	14.3964848689	19.9359625191	5.8615231274
N	14.5600323744	10.9684220006	10.8496001290
N	20.0919365756	10.8923378335	5.6806885751
N	8.8358001745	20.0131881944	11.1075388534
N	8.9163978020	3.1461955220	10.8523479541
N	20.0104303647	5.2650298128	5.9468476413
N	15.4069410614	4.4535331259	10.8358267201
N	13.5305172148	4.0424581181	5.9514358033
N	14.3961808567	8.7356881794	5.8683917940
N	14.5644491035	-0.3235949652	10.8542282652
N	20.1004897948	-0.2914431273	5.6969488793
N	8.8418605677	8.8040543285	11.1110221255
N	-2.1847558987	14.3437299810	10.8402009298
N	8.8116643523	16.4721877994	5.9496699454

N	12.4947796611	8.0738271726	2.6414306248
N	18.3616102201	2.1514968331	19.0865631246
N	10.8842655974	9.6719727666	13.6123621863
N	14.0125681529	5.0601420159	19.0908007139
N	15.4333804059	6.5448054214	13.6188274987
N	20.0135368775	16.8319493687	30.0729550075
N	9.4462422059	12.4533173959	2.7230680392
N	16.9028416245	12.4618281197	30.0702655818
N	12.5509204188	16.8137549686	2.6416731110
N	18.4172995008	10.8892125204	19.0848204734
N	11.0476222909	18.3871900281	13.7108344540
N	14.0614283980	13.9835449637	19.0877999485
N	15.3945080755	15.3063767352	13.6990306978
C	3.0357987211	7.9242243638	27.1626777406
C	8.8256100289	3.8618184207	5.6284305682
C	7.3949636741	3.5553327492	27.1674605100
C	4.4859569578	8.2420438161	5.5436584112
C	6.3115486148	6.1170091073	19.6820988283
C	5.6442941221	5.6560703587	13.1515957431
C	3.3563609540	7.0374490500	29.4985092150
C	8.5368478461	4.7811613384	3.3042303306
C	5.3628405959	5.8680549857	21.9994953942
C	6.5197862976	5.8876586088	10.7989705807
C	9.7560005421	10.3196532425	22.0213343516
C	2.0691123057	1.5444572002	10.8018232011
C	4.6563593891	4.7439539049	22.4162856732
C	7.2021563294	7.0234267694	10.3724651179
C	6.5775798452	5.7274799420	21.1251457350
C	5.3289875953	6.0082370554	11.7087347260
C	2.2791370312	10.3728778105	21.0962800542
C	9.5872359915	1.3967456107	11.6973395059
C	3.7791970021	7.2632829842	23.1780737571
C	8.0827179471	4.5218305177	9.5582902740
C	8.0084469881	2.5407734146	23.2608719808
C	3.8573076303	9.2613797748	9.4689110506
C	7.1941442881	2.6846427477	29.5228231937
C	4.6919471761	9.0965801800	3.1826951508
C	1.9923273951	10.0035827578	19.6522804060
C	9.8837638684	1.7849999915	13.1346507742
C	4.2661019150	8.5181062113	26.8854048913
C	7.5768014687	3.2972655671	5.8838320568
C	9.2496366573	9.0520821734	22.3020551957
C	2.6026355317	2.7973404828	10.5059985379
C	6.1344995290	4.0381490319	26.8153906554
C	5.7420969657	7.7533041866	5.9032022994
C	2.9564716141	6.7259671732	28.0673268637
C	8.9414581923	5.0686654620	4.7390954167
C	10.6222489838	8.4867118838	26.6203019935
C	1.2197268379	3.2761440815	6.1818202268
C	7.1385191152	5.8116721498	25.5386928284
C	4.7219836313	5.9932961916	7.1860159032
C	3.1782584119	10.2029056268	25.5516090427
C	8.6033750054	1.5631998692	7.2002834492
C	7.5876785694	11.1104476390	28.0754301270
C	4.3043227138	0.6856903548	4.6288892852
C	8.5233435269	4.2174538060	26.6926957629
C	3.3515190588	7.5895085528	6.0174101947
C	4.9083754268	7.1293699082	22.3828618641
C	6.9747278096	4.6350418135	10.3861074479
C	8.3956053232	5.3431393231	25.8880259898
C	3.4692455999	6.4673661312	6.8285948789
C	6.0059612050	5.1529335597	25.9996077415
C	5.8602677137	6.6426183850	6.7259063987
C	3.0758395590	6.1351106632	23.5801234568
C	8.7638357386	5.6615875514	9.1506493364
C	9.1326032450	2.6711798994	22.4522968845
C	2.7352710762	9.1323994881	10.2805072709
C	3.5129557362	4.8776723531	23.1945515542
C	8.3239089794	6.9104188264	9.5602289924
C	7.4711164709	10.0251765709	23.5996406158
C	4.3369571514	1.7722642425	9.1874496408
C	8.1148681311	8.9074184044	23.0838547238
C	3.7295473922	2.9085365261	9.7065060436
C	4.3335896143	9.6530256871	26.0919944466
C	7.4687796873	2.1528519178	6.6579326149
C	10.6904075378	9.6149229438	25.8104312240
C	1.1152414507	2.1392461054	6.9765412794
C	3.0977708624	16.6618526799	27.1626378473
C	8.8788139950	12.6106330720	5.6288703928
C	7.4504407321	12.2991524525	27.1658242708
C	4.5397829768	16.9805967449	5.5452256345
C	6.3786221203	14.8536327257	19.6720498905
C	5.6134501697	14.4272270754	13.0735149470
C	3.4141314302	15.7784316887	29.5005383990
C	8.5881071309	13.5205630829	3.3005975338
C	5.4381037578	14.6122025850	21.9928333742
C	6.5565087621	14.6789199021	10.7508926157
C	9.8787722808	19.0291050400	21.9270948146
C	2.0942583289	10.2555302195	10.7973699651
C	4.7242226588	13.4877472121	22.3962498935
C	7.2828552651	15.7976554365	10.3535757772
C	6.6512273337	14.4736656038	21.1163944406
C	5.3473372736	14.8237100020	11.6320292396
C	2.3312959923	19.1294329825	21.1040657075
C	9.5997401148	10.1049045276	11.5471928060
C	3.8564130263	16.0045456635	23.1766772165
C	8.1103590065	13.2780141028	9.5406385805
C	7.9714614314	11.2914566134	23.3351994043
C	3.9206141751	17.9997172862	9.4686891601
C	7.2481384177	11.4266077609	29.5211625862
C	4.7455135176	17.8364591661	3.1841266384
C	2.0468922673	18.7520676050	19.6617300745
C	9.8248325348	10.5134028808	12.9914260382
C	4.3299743283	17.2490695919	26.8788851109
C	7.6325328016	12.0411127350	5.8860884728
C	9.3706714445	17.7661402917	22.2255003165
C	2.5967114232	11.5183641491	10.4890733701
C	6.1904169696	12.7823020967	26.8127072691
C	5.7965531608	16.4937248223	5.9052647203
C	3.0168304960	15.4650563045	28.0690604285
C	8.9906920541	13.8142040773	4.7347650723
C	10.6799460103	17.2244011950	26.6200294728
C	1.2716588040	12.0103960246	6.1754381403
C	7.1957215639	14.5521453359	25.5315232261
C	4.7786146630	14.7348108564	7.1911645970
C	3.2448597113	18.9354490749	25.5455817566
C	8.6669114526	10.3185907958	7.2112063393
C	7.6426783124	19.8465264735	28.0772013909
C	4.3621885593	9.4269914357	4.6274949431

N	12.4937994667	8.0739863925	2.6419634992
N	18.3530018939	2.1433532460	18.9951655624
N	10.8855314414	9.6627259646	13.6194768725
N	14.0019138595	5.2244630713	18.9979602975
N	15.4252878736	6.5512997330	13.6257559720
N	20.0133941935	16.8317659984	30.0723545766
N	9.4469903041	12.4538027399	2.7226323859
N	16.9032504644	12.4621312521	30.0708611274
N	12.5488995132	16.8146289881	2.6418999388
N	18.4069024891	10.8822625395	18.9953566809
N	11.0427054496	18.3923006761	13.7176522947
N	14.0513423440	13.9700371315	18.9925676892
N	15.3964178526	15.3056022866	13.7160704469
C	3.0400272137	7.9237081502	27.1648393397
C	8.8193616456	3.8750990011	5.6145496239
C	7.3959554160	3.5634228757	27.1695768515
C	4.4692854495	8.2339654775	5.5468808904
C	6.3235716040	6.1021703948	19.5641165391
C	5.6577319553	5.6457207439	13.1730843269
C	3.3549816872	7.0342553773	29.5015338241
C	8.5393874800	4.7907575032	3.2894357260
C	5.4084791062	5.8655623042	21.9035110625
C	6.4972648068	5.8540966725	10.8055353510
C	9.8317444107	10.2933142129	21.9912234854
C	2.0407837230	1.4939977536	10.8074879283
C	4.6980358468	4.7426337062	22.3193551525
C	7.2000369915	6.9717864898	10.3650482423
C	6.6088039607	5.7244337792	21.0076544364
C	5.3254739112	6.0026039235	11.7350263342
C	2.2926901106	10.3732007223	20.9955667617
C	9.5596167558	1.4380075575	11.7288833333
C	3.8526177937	7.2596261117	23.1220000469
C	8.0133779276	4.4475093366	9.5539652915
C	8.0049226337	2.5559213580	23.2629488049
C	3.8438246035	9.2684890628	9.4698182626
C	7.1982848219	2.6820902179	29.5224555821
C	4.6681573569	9.1117313354	3.1913603240
C	1.9827846985	9.9960560969	19.5579837795
C	9.8783980061	1.8057720246	13.1666891830
C	4.2709926828	8.5164410525	26.8870602089
C	7.5737855434	3.2956858687	5.8517323188
C	9.3193921024	9.0275186959	22.2706330869
C	2.5480211362	2.7605760563	10.5222690464
C	6.1349993165	4.0450204377	26.8177081221
C	5.7300070252	7.7456644228	5.8901077205
C	2.9590634429	6.7249567113	28.0690478577
C	8.9347832474	5.0820365493	4.7256686197
C	10.6286970923	8.4865452150	26.6195792575
C	1.2379447349	3.3156699408	6.1757103231
C	7.1375203904	5.8146230075	25.5336654884
C	4.7273777334	5.9918855516	7.1963170599
C	3.1847710369	10.1955475231	25.5449037725
C	8.6021624492	1.5786876848	7.1883454946
C	7.5912698247	11.1132808500	28.0743537301
C	4.2708750414	0.6834752545	4.6384485456
C	8.5235998694	4.2253419579	26.6929495656
C	3.3418351803	7.5828452489	6.0386004876
C	4.9692443240	7.1260085748	22.3086935926
C	6.9185083810	4.5896676499	10.3943403363
C	8.3951230248	5.3481317846	25.8840884752
C	3.4700130235	6.4650272574	6.8544209010
C	6.0056312906	5.1574995569	25.9985329759
C	5.8590127040	6.6376723785	6.7152909250
C	3.1463719847	6.1327443115	23.5232231475
C	8.7137356441	5.5693044371	9.1291620142
C	9.1229841174	2.7146677052	22.4505781305
C	2.7208454890	9.1392244585	10.2804358019
C	3.5675289054	4.8761425641	23.1167513370
C	8.3084372972	6.8296778316	9.5389753614
C	7.5491720226	10.0088821106	23.5759642524
C	4.3039819298	1.7845570476	9.1943645165
C	8.1850356411	8.8887720569	23.0551527055
C	3.6719705385	2.9030527634	9.7235264517
C	4.3398207731	9.6478829459	26.0882918546
C	7.4681539104	2.1523060937	6.6281805907
C	10.6958310058	9.6123939189	25.8062069876
C	1.1593616120	2.1814980712	6.9763259824
C	3.0959666382	16.6661511376	27.1642977950
C	8.8881742335	12.6180107621	5.6199969956
C	7.4548496177	12.3052134801	27.1683030678
C	4.5219626917	16.9745265471	5.5482335179
C	6.3515491887	14.8540090712	19.5912101130
C	5.6188239321	14.4280431972	13.1143615339
C	3.4115517185	15.7756320828	29.5007658766
C	8.5803386021	13.5194129651	3.2906266700
C	5.4393869685	14.6041224916	21.9297694448
C	6.5342091833	14.6776673030	10.7766667361
C	9.8765743045	19.0890058719	21.8996477069
C	2.0836524617	10.2614778241	10.8041626069
C	4.7250340813	13.4845772395	22.3465140253
C	7.2526388400	15.7967885110	10.3660063279
C	6.6346250066	14.4584811389	21.0294655554
C	5.3380080674	14.8235863488	11.6755845464
C	2.3412659803	19.1523667348	21.0098487445
C	9.5825759468	10.1627795337	11.5790309576
C	3.9007934403	16.0053573353	23.1599514474
C	8.0686142855	13.2774207915	9.5400988146
C	8.0575056982	11.2729685602	23.3153748364
C	3.9115991527	17.9991520429	9.4618762776
C	7.2524842611	11.4235871977	29.5211823391
C	4.7213262114	17.8519849491	3.1923588028
C	2.0472423575	18.7577143484	19.5739283107
C	9.8399379191	10.5387885972	13.0269306390
C	4.3266207008	17.2581459817	26.8833955413
C	7.6564924150	12.0179728105	5.8779771110
C	9.3822595280	17.8156446926	22.1769726714
C	2.5911215527	11.5242656966	10.5031827385
C	6.1954948753	12.7902526045	26.8157060345
C	5.7834131426	16.4888683842	5.8926753596
C	3.0167174731	15.4667074005	28.0678663709
C	8.9750172258	13.8227381575	4.7247468162
C	10.6804644527	17.2248756624	26.6208369434
C	1.2679574569	12.0400888047	6.1865362450
C	7.2034674263	14.5541146911	25.5274777298
C	4.7831094926	14.7345047980	7.1994311013
C	3.2382266020	18.9376696650	25.5434206379
C	8.7362253450	10.3236633404	7.2053094956
C	7.6454898708	19.8513018466	28.0752560451
C	4.3300831490	9.4234742240	4.6380117500

N	4.2835258912	15.6526965357	10.8392206479
N	2.4125475116	15.2423287914	5.9488425776
N	3.2720176774	19.9408783046	5.8692784719
N	3.4435987110	10.8882275230	10.8441421577
N	8.9715005626	10.8936509108	5.6828339088
N	-2.2700075490	20.1920929179	11.1069367179
N	-2.0872592489	3.0546492137	10.7526153860
N	8.8882153246	5.2746190920	5.9455332905
N	4.2946158717	4.4440484737	10.8345102037
N	2.4164922109	3.9482557242	5.9476423586
N	3.2774054532	8.7339244323	5.8689957233
N	3.4355070978	-0.2159108727	10.8306777091
N	8.9413981950	-0.7775918234	5.7772059674
N	-2.2678401956	8.7995133354	11.1102179160
C	8.9440059908	15.7183880813	11.4099323586
C	19.9833764510	15.1099526130	5.3702806562
C	8.3900169563	15.7533413890	12.8203044797
C	20.5481575342	15.0884224942	3.9639346375
C	8.5473731261	13.7587509454	14.3482474996
C	20.3848889788	17.0838516457	2.4390821101
C	9.2465806540	12.9547919217	15.2403986289
C	19.6923855396	17.8758201678	1.5310037808
C	10.5530999546	13.2694732626	15.5830346118
C	18.3997323011	17.5384964932	1.1580849245
C	11.1518456814	14.3992750584	15.0405551676
C	17.8075377445	16.4002175693	1.6907775734
C	10.4492275907	15.2032717205	14.1550791463
C	18.5023747184	15.6094276837	2.5947951026
C	9.1405135886	14.8895642128	13.7933291342
C	19.7987605693	15.9436082351	2.9822689310
C	14.0578497021	15.5504725922	11.3350340369
C	14.8895787751	15.3760597782	5.3543883532
C	14.0866151334	14.9904722324	12.7444775348
C	14.8227804212	15.9286692128	3.9436388292
C	15.9908024661	15.6537855482	14.2464856946
C	12.8843229769	15.2499175200	2.4930094899
C	16.5886804567	16.5125294693	15.1612781507
C	12.2657598139	14.3835995981	1.5996476447
C	15.9057116166	17.6326811037	15.6121168081
C	12.9414540061	13.2627554248	1.1392761651
C	14.6266461022	17.8921612063	15.1347572486
C	14.2349119368	13.0118044742	1.5815643393
C	14.0403771423	17.0443257669	14.2057818550
C	14.8412025184	13.8684890031	2.4896822592
C	14.7157526408	15.9105393748	13.7528107546
C	14.1730360294	15.0016463298	2.9549389206
C	14.4363672911	21.2956468814	5.2571306770
C	14.5090018876	9.5903739575	11.4103726741
C	15.1124568412	21.2790927096	3.9010038069
C	13.8432342485	9.5702523119	12.7721872632
C	13.1720251279	20.7862836006	2.3794442157
C	15.7838608306	10.0413480030	14.2994209043
C	12.5029614914	19.9743201123	1.4749355760
C	16.4557542806	10.8457256169	15.2088136421
C	13.0775460791	18.7823962323	1.0512296929
C	15.8861628956	12.0375519029	15.6401409344
C	14.3244106407	18.4156718955	1.5358330108
C	14.6429632866	12.4155392921	15.1546628865
C	14.9876839119	19.2244029419	2.4502821632
C	13.9764541151	11.6149176777	14.2355448479
C	14.4165485167	20.4171402847	2.8871349787
C	14.5414827697	10.4207804413	13.7942346584
C	20.2305258566	20.7313367071	5.1925168360
C	8.6928062051	10.1675912365	11.6741648419
C	20.7581864237	20.7320475189	3.7702205219
C	8.1964414914	10.0981320297	13.1055831695
C	18.6287414050	21.1436881677	2.4939911968
C	10.3739816197	9.6690295434	14.2919522961
C	17.8276760880	21.9322682483	1.6805795131
C	11.2006697341	8.8746023118	15.0733405117
C	18.3037345799	11.9451925281	1.1686344154
C	10.7778449995	18.9450192374	15.5757836572
C	19.5921075734	12.3538529247	1.4824983440
C	9.5053532497	18.4718210409	15.2896513152
C	20.3947995719	11.5640753325	2.2958333182
C	8.6402578778	19.2297174663	14.5103331441
C	19.9167904995	21.5516449316	2.8342320551
C	9.0731225010	9.2665733417	13.9977472883
C	8.9501604334	4.5184014530	11.4250861581
C	19.9960046762	3.9008379348	5.3576561727
C	8.3926757776	4.5492306731	12.8340663577
C	20.5602557471	3.8942646803	3.9513401329
C	8.5430997001	2.5473448737	14.3528473245
C	20.3916032017	5.8834422970	2.4197817978
C	9.2342604822	1.7465826385	15.2539524702
C	19.6965357870	6.6729350993	1.5116066982
C	10.5361547711	2.0640992014	15.6123611000
C	18.3985094408	6.3410234745	1.1527962542
C	11.1379171485	3.1950710643	15.0752892864
C	17.8055364324	5.2079607158	1.6954577403
C	10.4435988828	3.9970755401	14.1807867508
C	18.5044922766	4.4177014148	2.5969533584
C	9.1384405004	3.6819245131	13.8076278185
C	19.8053423249	4.7478374323	2.9729035937
C	14.0346193008	4.3341837721	11.3855762973
C	14.9078415556	4.1664799543	5.4065974302
C	14.0551662193	3.7585319921	12.7883480851
C	14.8861748731	4.7139359983	3.9926493860
C	16.0168062427	4.3826797656	14.2285759232
C	12.9493236471	4.0409955408	2.5375005394
C	16.6641877822	5.2344339026	15.1155974611
C	12.3322582101	3.1765639256	1.6406452348
C	16.0148471000	6.3652760291	15.5891293123
C	13.0089204795	2.0566964950	1.1784673141
C	14.7204714882	6.6424634722	15.1656399504
C	14.3001760750	1.8023291073	1.6255365402
C	14.0844431392	5.8010068681	14.2645028895
C	14.9050768146	2.6554706384	2.5376425346
C	14.7264860707	4.6585065779	13.7866723737
C	14.2377850322	3.7902159515	3.0003363233
C	14.4507391252	10.1137121106	5.3088068452
C	14.4808775121	-1.6808186017	11.4563403782
C	15.0854617455	10.1239715221	3.9320784352
C	13.7958110489	-1.6465790587	12.8075727632
C	13.0973068214	9.6595683300	2.4619886867
C	15.7481531922	-1.1766973922	14.3220433993
C	12.3963298982	8.8553796767	1.5747275156
C	16.4233908458	-0.3761374922	15.2318505844
C	12.9503900875	7.6612173190	1.1295669374

C	8.5791890791	12.9601272861	26.6904448704
C	3.4061600660	16.3274959066	6.0201700009
C	4.9907266331	15.8722220712	22.3884669488
C	6.9847959207	13.4172006702	10.3398914120
C	8.4524848073	14.0839273619	25.8829291148
C	3.5251975949	15.2065958561	6.8330004843
C	6.0626126973	13.8951036865	25.9939427446
C	5.9162123950	15.3848151056	6.7302170885
C	3.1431825976	14.8765147430	23.5617787441
C	8.8361315310	14.4001755370	9.1616387756
C	9.1058654829	11.4346004032	22.5433287383
C	2.7947585549	17.8901913308	10.2778202902
C	3.5774238670	13.6200219814	23.1695057588
C	8.4229446755	15.6582602910	9.5709020941
C	7.6154040546	18.7587575287	23.5420532345
C	4.3539027089	10.5231105289	9.1769293393
C	8.2470920839	17.6340103579	23.0261534263
C	3.7187908563	11.6490266324	9.6858675384
C	4.4003844561	18.3807557644	26.0810215112
C	7.5294973494	10.8997126666	6.6655646064
C	10.7492290872	18.3525505713	25.8102610767
C	1.1640226980	10.8691675440	6.9633547227
C	11.7755604354	7.9234785384	27.1609748754
C	17.5640843171	3.8677201873	5.6300056433
C	16.1347174894	3.5569702423	27.1673922840
C	13.2145311407	8.2403315584	5.5476088692
C	15.0152132835	6.0132218299	19.6460267346
C	14.3585829709	5.6760600563	13.0686847922
C	12.0956764769	7.0380774251	29.4979171884
C	17.2737326134	4.7805524400	3.3025718877
C	14.0801145709	5.8600119335	21.9840771617
C	15.2585296319	5.9147679443	10.7362630548
C	18.5444086550	10.2569123683	21.9776518263
C	10.7842111035	1.5268163878	10.7965160882
C	13.3718376647	4.7385052739	22.4055292411
C	15.9665987186	7.0441530159	10.3352964053
C	15.2929691351	5.7143716933	21.1085536506
C	14.0542474573	6.0437311595	11.6262025112
C	10.9725400842	10.4678452054	21.1515296833
C	18.3406844604	1.4285338665	11.6921178846
C	12.5060820928	7.2618551947	23.1675939116
C	16.8385753335	4.5336137714	9.5369887538
C	16.7432334190	2.5413751145	23.3235409532
C	12.6468413169	9.2601793726	9.4359109005
C	15.9330400247	2.6851622202	29.5222381287
C	13.4179183477	9.1036502108	3.1885568627
C	10.7259576180	10.0571606028	19.7110550803
C	18.6302423761	1.8028034526	13.1341862033
C	13.0070010044	8.5128785607	26.8788656930
C	16.3169953402	3.2993256484	5.8856909267
C	18.0101406791	9.0041129950	22.2724742956
C	11.2877821918	2.7863071737	10.4770042316
C	14.8744759644	4.0384727350	26.8128108743
C	14.4739942262	7.7559901467	5.9013795363
C	11.6952096987	6.7262459846	28.0669601131
C	17.6777390208	5.0718997686	4.7367821833
C	19.3605447188	8.4839679155	26.6183303254
C	9.9579050986	3.2636466762	6.1760244797
C	15.8791754510	5.8131345441	25.5381604548
C	13.4660848593	5.9934966730	7.1904589751
C	11.9202451866	10.1998746716	25.5470545004
C	17.3479270201	1.5752963273	7.2120057236
C	16.3272678347	11.1079448207	28.0764180628
C	13.0492192820	0.6856971788	4.6277972006
C	17.2633384774	4.2204216353	26.6950912706
C	12.0847800801	7.5841488162	6.0274858105
C	13.6321904072	7.1229901671	22.3689363685
C	15.7091266062	4.6581140107	10.3330659243
C	17.1360941272	5.3461160855	25.8904092965
C	12.2099454002	6.4630056177	6.8389668369
C	14.7463148040	5.1530692155	25.9965932626
C	14.5999774755	6.6464162260	6.7245952567
C	11.7991367968	6.1366480342	23.5711391854
C	17.5447059564	5.6663967818	9.1535253369
C	17.8727452767	2.6562496993	22.5203280610
C	11.4882314838	9.1284168118	10.1941634705
C	12.2301831269	4.8767959578	23.1857198970
C	17.1101279349	6.9197059296	9.5551709029
C	16.2540812064	10.0312729631	23.5602092074
C	13.0433381609	1.7788745256	9.1720405574
C	16.8725843979	8.8939705056	23.0569636050
C	12.4097613580	2.9094498541	9.6723168195
C	13.0759395028	9.6459400304	26.0828311070
C	16.2119903492	2.1579101042	6.6648248307
C	19.4276489586	9.6130888238	25.8096752236
C	9.8503767054	2.1233243096	6.9652999738
C	11.8324972053	16.6619045782	27.1629050819
C	17.6177759549	12.6068454502	5.6252070679
C	16.1893573437	12.2961418901	27.1662823797
C	13.2813673864	16.9808214169	5.5448086429
C	15.1192241207	14.8488424126	19.6766066150
C	14.3603349997	14.4300372742	13.0833358875
C	12.1507004508	15.7770518796	29.4996326624
C	17.3314465637	13.5237165448	3.3003687495
C	14.1717814197	14.6091798560	21.9954808974
C	15.3161283607	14.6762686028	10.7598366369
C	18.6010554033	19.0057651243	21.9911508237
C	10.8359603117	10.2483626072	10.7037188771
C	13.4586221712	13.4869218627	22.4060926556
C	16.0358054194	15.7953561251	10.3515605853
C	15.3862927307	14.4662251382	21.1214595260
C	14.1053159742	14.8233106549	11.6387803994
C	11.0757719463	19.1666324075	21.0284140289
C	18.3745225368	10.1177214560	11.6962911606
C	12.5879986215	16.0075529799	23.1696172540
C	16.8733143172	13.2735942919	9.5536879179
C	16.7812855600	11.2833675299	23.2802997850
C	12.6544491168	17.9999010025	9.4736247902
C	15.9878233381	11.4253645514	29.5219302115
C	13.4882059562	17.8341164612	3.1830514388
C	10.7724305581	18.8048336876	19.5862672831
C	18.6667418489	10.5131977592	13.1327106168
C	13.0642383802	17.2514349664	26.8824351208
C	16.3692916075	12.0394993402	5.8758287568
C	18.0668235873	17.7508761680	22.2768780115
C	11.3635608503	11.5115977821	10.4418836738
C	14.9288939082	12.7784931918	26.8136015103
C	14.5370263784	16.4920648792	5.9062216787
C	11.7513702640	15.4645538811	28.0685213252

C	8.5844102976	12.9629763037	26.6904483769
C	3.3953911060	16.3219698938	6.0401205771
C	5.0121198448	15.8666367033	22.3407937621
C	6.9577020096	13.4159073444	10.3599090833
C	8.4599370034	14.0840654939	25.8785259766
C	3.5249727525	15.2048681523	6.8566815849
C	6.0694213196	13.9018905762	25.9946836489
C	5.9139866073	15.3821413905	6.7193138002
C	3.1903740343	14.8816565686	23.5626094639
C	8.7834298168	14.4006247439	9.1442036178
C	9.1913587646	11.4103117374	22.5215506369
C	2.7874962191	17.8584153608	10.2692327586
C	3.6009931555	13.6231294195	23.1517849998
C	8.3772574149	15.6583065985	9.5617237848
C	7.6265306171	18.7636544136	23.5244052177
C	4.3448950313	10.5300345619	9.1844974966
C	8.2654723841	17.6554514542	22.9825210180
C	3.7143776575	11.6550105846	9.7013039787
C	4.3944450975	18.3890241737	26.0835794132
C	7.5841985080	10.8743027494	6.6586334761
C	10.7479986647	18.3510945315	25.8078798031
C	1.1639048434	10.9028434152	6.9804866024
C	11.7819996927	7.9282582359	27.1649169868
C	17.5696846428	3.8806867484	5.6229024192
C	16.1373867976	3.5624015288	27.1692485138
C	13.2063211444	8.2344422641	5.5490885135
C	15.0284191903	6.1306004149	19.5808770401
C	14.3251072280	5.7092484454	13.0847854498
C	12.0961146682	7.0365300402	29.5011601810
C	17.2654699425	4.7800079087	3.2918004742
C	14.1261763153	5.8877153843	21.9297728973
C	15.2155720484	5.9332793075	10.7458757152
C	18.5787843177	10.2522542701	21.9036653614
C	10.7481963946	1.5523766415	10.8161390353
C	13.4255697171	4.7600188852	22.3471705439
C	15.9362851957	7.0515971057	10.3360619350
C	15.3194640210	5.7596546030	21.0243854263
C	14.0207965500	6.0844931229	11.6443341174
C	11.0317989265	10.4412710491	21.0983979388
C	18.3246179240	1.3457527995	11.7085845110
C	12.5817988919	7.2699939903	23.1733051160
C	16.7705139215	4.5279400463	9.5431670341
C	16.7555881581	2.5318719620	23.2455499112
C	12.5972353387	9.2631234690	9.4457983632
C	15.9379636547	2.6816385379	29.5222266081
C	13.4056985981	9.1117446580	3.1928573792
C	10.7441833300	10.0490735065	19.6603561411
C	18.6227642837	1.7077593092	13.1525076611
C	13.0127243059	8.5201114648	26.8839898771
C	16.3346117012	3.2888307136	5.8840835464
C	18.0554976290	8.9970814143	22.2087740099
C	11.2785049045	2.8044647592	10.5112598604
C	14.8767292476	4.0476825935	26.8214745259
C	14.4676553123	7.7477270848	5.8924832406
C	11.7021295393	6.7283977031	28.0678950627
C	17.6617654816	5.0833689995	4.7254538010
C	19.3655691188	8.4808000974	26.6164927453
C	9.9504496682	3.2942283268	6.1820588092
C	15.8804023171	5.8121706732	25.5311043963
C	13.4666576079	5.9949457055	7.2008645674
C	11.9244679688	10.1999226170	25.5440601080
C	17.4051628365	1.5916915912	7.2148017048
C	16.3299287019	11.1119769559	28.0752875798
C	13.0148902331	0.6859870053	4.6369693539
C	17.2653784739	4.2201428560	26.6876252581
C	12.0795561876	7.5835838180	6.0428593963
C	13.6898627877	7.1445832126	22.3479286383
C	15.6475149596	4.6692934101	10.3455074930
C	17.1379246800	5.3420455691	25.8772571802
C	12.2085609007	6.4668221461	6.8599694763
C	14.7480843808	5.1596400047	26.0015370468
C	14.5978261657	6.6409476638	6.7191488316
C	11.8836646942	6.1385160064	23.5750468262
C	17.4883179510	5.6498741757	9.1494193204
C	17.8815316721	2.6583918138	22.4384943929
C	11.4430504610	9.1507276892	10.2135279012
C	12.3038983552	4.8854722517	23.1577160458
C	17.0729245559	6.9101975163	9.5494756345
C	16.3196127768	10.0165781389	23.5310012867
C	12.9969280604	1.7755811883	9.1744848721
C	16.9332036514	8.8822636732	23.0143447409
C	12.3957547742	2.9131431712	9.6980104889
C	13.0806401416	9.6510947979	26.0842094583
C	16.2558037583	2.1484412986	6.6685288670
C	19.4340287680	9.6065533995	25.8030179200
C	9.8452931272	2.1554164245	6.9736941320
C	11.8337165924	16.6658496624	27.1655319176
C	17.6142670297	12.6182957593	5.6175159403
C	16.1904240784	12.3036517632	27.1693567755
C	13.2637444591	16.9764870839	5.5461722267
C	15.1032141199	14.8595603154	19.5514783463
C	14.3547692018	14.4278595382	13.1174992436
C	12.1493668852	15.7752950522	29.5016332461
C	17.3325067859	13.5301041039	3.2903431861
C	14.1826489336	14.6993250403	21.8971967253
C	15.2772056686	14.6721005618	10.7810865191
C	18.6240959993	19.0147388625	21.9116692647
C	10.8090355122	10.2830963254	10.7188689113
C	13.4729107831	13.5951609161	22.3605696320
C	15.9913440519	15.7906116308	10.3618078045
C	15.3805616153	14.5216815736	21.0053651613
C	14.0794291457	14.8201564602	11.6770715382
C	11.0651826073	19.2564733623	20.9943033254
C	18.3672478873	10.1215584303	11.7074826631
C	12.6418528103	16.1429978335	23.0735606513
C	16.8167570968	13.2676441808	9.5556462110
C	16.8376480420	11.2707320078	23.2432770466
C	12.5829429580	18.0039141479	9.4643494356
C	15.9923874780	11.4221056077	29.5224520500
C	13.4624136168	17.8524095123	3.1904675955
C	10.7647730747	18.9071661209	19.5478981996
C	18.6644370755	10.5102838103	13.1449503296
C	13.0647190550	17.2568673186	26.8842650294
C	16.3693851437	12.0373811136	5.8549104326
C	18.1105627134	17.7533235644	22.2086244755
C	11.3504453968	11.5373100510	10.4436933644
C	14.9296069446	12.7893491921	26.8225381610
C	14.5245402348	16.4908680989	5.8930349099
C	11.7536429795	15.4664484103	28.0690180700

C	15.8610370952	0.8184828179	15.6640761596
C	14.2108463954	7.2845815366	1.5700029566
C	14.6180304406	1.2008855285	15.1826555464
C	14.9051872738	8.0852154481	2.4683145732
C	13.9471817721	0.4023853173	14.2648711549
C	14.3546815562	9.2790878121	2.9281909730
C	14.5054976804	-0.7937162845	13.8205978688
C	20.2383006227	9.5123581409	5.1552272953
C	8.6183661671	-1.0491818322	11.6883389957
C	20.7694242078	9.5387027552	3.7351596097
C	8.1175328872	-1.1581320034	13.1150356809
C	18.6295400916	9.9508999467	2.4751895057
C	10.3113986800	-1.4736445573	14.3090118160
C	17.8246941498	10.7398822519	1.6657175015
C	11.1771039206	-2.2277463855	15.0880531747
C	18.3071594581	0.7300370554	1.1903529639
C	10.7338266585	7.6673905656	15.5771528351
C	19.5920720777	1.1409020006	1.5153054563
C	9.4364476865	7.2615618527	15.2984559590
C	20.3912409583	0.3515015858	2.3328185938
C	8.6124477017	8.0571121598	14.5123544320
C	19.9211991856	10.3571966460	2.8045323258
C	9.0351422782	-1.9437915565	14.0062877870
C	-2.1635754942	15.7219765246	11.3948740724
C	8.8503601803	15.1052275794	5.3702263733
C	-2.7177158029	15.7650876836	12.8052741026
C	9.4367804805	15.1052281054	3.9731473816
C	-2.5657215868	13.7644138393	14.3294373452
C	9.2635811608	17.0892784180	2.4369923141
C	-1.8720532881	12.9630314577	15.2282580825
C	8.5664245148	17.8748447366	1.5273242135
C	-0.5706110018	13.2828849241	15.5854468298
C	7.2705445426	17.5372964965	1.1662454028
C	0.0284333571	14.4153479349	15.0487372324
C	6.6814095343	16.4021506762	1.7084263963
C	-0.6676362741	15.2160713433	14.1546741880
C	7.3819964665	15.6150502442	2.6113622216
C	-1.9726685316	14.8995021794	13.7822847529
C	8.6808322006	15.9512190880	2.9885673498
C	2.9102636958	15.4905529709	11.3804109632
C	3.7646802667	15.3812967731	5.3521611931
C	2.9405570568	14.9179174165	12.7833809632
C	3.6931722899	15.9372159170	3.9426388500
C	4.8658304927	15.5690861202	14.2608028519
C	1.7680929213	15.2599211259	2.4740110295
C	5.4866250048	16.4332259233	15.1545026699
C	1.1554623063	14.3905466116	1.5796649762
C	4.8214180692	17.5663335432	15.5997848074
C	1.8298573659	13.2628416500	1.1346769020
C	3.5373470855	17.8338703696	15.1400222019
C	3.1159808494	13.0065620952	1.5950864523
C	2.9278768835	16.9791420897	14.2329393777
C	3.7167324669	13.8667182908	2.5034682119
C	3.5853808875	15.8331169526	13.7846484329
C	3.0493740306	15.0066371238	2.9531847172
C	3.3331377695	21.3233593235	5.3239691527
C	3.3760626072	9.5220395499	11.4294642185
C	3.9641774774	21.3413677088	3.9459379833
C	2.7043785929	9.5414747177	12.7874913415
C	1.9728881143	20.8737913522	2.4809125130
C	4.6714899686	10.0264323193	14.2780304467
C	1.2739020470	20.0713542430	1.5903344245
C	5.3485380120	10.8283599505	15.1856570033
C	1.8318101929	18.8822970904	1.1358733998
C	4.7712671412	12.0070480775	15.6419073860
C	3.0943999784	18.5074335614	1.5717665494
C	3.5147998783	12.3743619003	15.1832269678
C	3.7869494037	19.3062921292	2.4732686421
C	2.8438815483	11.5769387107	14.2640295459
C	3.2336141765	20.4962729496	2.9409695312
C	3.4150691720	10.3948240858	13.7994921639
C	9.1791852794	20.4210526262	4.9523821622
C	-2.4291247604	10.1484306006	11.7051891674
C	9.7109524381	20.7828417165	3.5831207206
C	-2.9232245577	10.0443887626	13.1346155509
C	7.4742097805	21.1523813027	2.4864515536
C	-0.7323183897	9.6719816701	14.3151666160
C	6.6000714370	21.9210190226	1.7317448862
C	0.1185805868	8.8953100361	15.0879500641
C	7.1961309812	11.9481903837	1.1630232778
C	-0.1666827393	18.9425451007	15.6054527136
C	8.4832044713	12.3570979246	1.4818007387
C	-1.4467983610	18.4550763936	15.3864796678
C	9.2832930790	11.5667752420	2.2970050246
C	-2.3503751412	19.1949693467	14.6342148181
C	8.7677029556	21.5981492442	2.7458008803
C	-2.0200724982	9.2322984973	14.0175509983
C	-2.0849587804	4.4376576972	11.2965415675
C	8.8613173909	3.9019466277	5.3813457105
C	-2.6867260718	4.5013092354	12.6864917975
C	9.4237534245	3.8611717353	3.9746721943
C	-2.5398559659	2.5530559737	14.2741702131
C	9.2748709842	5.8563632063	2.4450786374
C	-1.8545234334	1.7905233337	15.2124496651
C	8.5814752338	6.6574487099	1.5456918271
C	-0.5677162841	2.1444900169	15.5901975346
C	7.2780439642	6.3403443987	1.1935490140
C	0.0238163447	3.2736596795	15.0383586237
C	6.6765706211	5.2112670510	1.7348013366
C	-0.6642303167	4.0357209088	14.1053807945
C	7.3724110510	4.4103448576	2.6285832299
C	-1.9527071255	3.6820843322	13.7095342912
C	8.6795288305	4.7245015834	2.9955189975
C	2.9198368060	4.2540097123	11.3609010546
C	3.7650947071	4.1317868053	5.3552797028
C	2.9504936229	3.6928093302	12.7687013946
C	3.6844275555	4.7157813260	3.9578504368
C	4.8647345679	4.3609463838	14.2529056453
C	1.7681892433	4.0516214579	2.4740344221
C	5.4759692689	5.2285054288	15.1498487446
C	1.1566936749	3.1875889672	1.5743333446
C	4.8036737349	6.3593554717	15.5902488489
C	1.8317348538	2.0625725304	1.1241651434
C	3.5224279010	6.6208764156	15.1202981292
C	3.1176224885	1.8044939520	1.5836325138
C	2.9225043573	5.7634621764	14.2087765404
C	3.7170852916	2.6598534334	2.4973462855
C	3.5862026126	4.6184899909	13.7672390050
C	3.0484222118	3.7959751934	2.9539225415

C	17.7340697535	13.8131372277	4.7354241044
C	19.4229408671	17.2255280259	26.6216844075
C	10.0137833804	12.0204935982	6.1793803505
C	15.9329177783	14.5498174614	25.5338844929
C	13.5147880063	14.7336959792	7.1893761098
C	11.9791964085	18.9377142870	25.5486959654
C	17.3953147592	10.3062663473	7.1934614483
C	16.3820381022	19.8477251450	28.0765674003
C	13.0801949934	9.4258199464	4.6333528934
C	17.3177112037	12.9576956023	26.6906335555
C	12.1461103695	16.3297126407	6.0186632909
C	13.7227797797	15.8711387587	22.3828224487
C	15.7500695174	13.4138901983	10.3562007416
C	17.1901301932	14.0821022819	25.8840725855
C	12.2624231086	15.2081914561	6.8309824806
C	14.8002936813	13.8918791306	25.9958717735
C	14.6540245702	15.3821096099	6.7301551158
C	11.8748622716	14.8815175305	23.5605200150
C	17.5908523261	14.3967287978	9.1627261494
C	17.9185828586	11.3919027224	22.4873035897
C	11.5329637558	17.8786752211	10.2871368785
C	12.3105613523	13.6229348236	23.1769553003
C	17.1728775563	15.6556152967	9.5647003823
C	16.3262259442	18.7668818238	23.5945510213
C	13.1685138534	10.5218464688	9.1909504216
C	16.9369215832	17.6344347558	23.0713455708
C	12.5223346763	11.6448080787	9.6926380493
C	13.1340664185	18.3845271764	26.0867001399
C	16.2610217448	10.8941092936	6.6484794655
C	19.4925051491	18.3527143248	25.8106397673
C	9.9113801404	10.8836219793	6.9741682973

C	17.7288284121	13.8233328621	4.7259374134
C	19.4206268597	17.2235379509	26.6180209083
C	10.0372648489	12.0581686984	6.1723673605
C	15.9326437873	14.5534501214	25.5306999317
C	13.5214609878	14.7325386140	7.1919742436
C	11.9768896927	18.9383670504	25.5466628836
C	17.3995121110	10.3245418565	7.1957369060
C	16.3862042906	19.8505062035	28.0753922548
C	13.0652342372	9.4228795101	4.6390293819
C	17.3181275117	12.9613266154	26.6870609930
C	12.1359995006	16.3226519352	6.0336322576
C	13.7508301993	15.9761867084	22.2566987955
C	15.7058174853	13.4087347547	10.3746344822
C	17.1900775015	14.0832324310	25.8768059524
C	12.2639910086	15.2037095801	6.8478515988
C	14.8006681563	13.9012162543	26.0024541553
C	14.6534438206	15.3818010455	6.7167576733
C	11.9382678441	15.0343096693	23.5269967721
C	17.5271964429	14.3903301869	9.1510119711
C	17.9598790187	11.3840159931	22.4289667616
C	11.4657475195	17.8967216631	10.2858939228
C	12.3516969739	13.7613807208	23.1650386659
C	17.1165944465	15.6497033199	9.5588858866
C	16.3560887349	18.7516200483	23.5232850801
C	13.1329564400	10.5162349501	9.1866427450
C	16.9836364759	17.6251325979	23.0058636397
C	12.5055136404	11.6508780939	9.6853712052
C	13.1329740369	18.3883028984	26.0853773957
C	16.2650611702	10.8948457341	6.6329139343
C	19.4876913887	18.3493652774	25.8044505538
C	9.9652097061	10.9215665380	6.9699231567

C	3.3802958852	10.1059361012	5.3039064132
C	3.3914808442	-1.5894907144	11.3996852200
C	4.0357669286	10.0885651339	3.9369619942
C	2.7163209663	-1.6033412156	12.7572635071
C	2.0365116061	9.6617075958	2.4709663040
C	4.6586892568	-1.1625934183	14.2935263114
C	1.3204127774	8.8722151572	1.5829949083
C	5.3317243304	-0.3757103151	15.2171416117
C	1.8528366323	7.6712112937	1.1305031201
C	4.7644291552	0.8103606401	15.6675258063
C	3.1084444400	7.2733590949	1.5649594564
C	3.5206660351	1.1975928932	15.1906075092
C	3.8193536474	8.0605226822	2.4622692728
C	2.8547942949	0.4152715568	14.2551772960
C	3.2903663716	9.2603265090	2.9298198857
C	3.4186400476	-0.7709278173	13.7915885195
C	9.1172777009	9.5145516911	5.1550007958
C	-2.4824022254	-0.9448797233	11.8407602842
C	9.6518530939	9.5405730881	3.7367700499
C	-2.9587785947	-1.2062952194	13.2538934758
C	7.5176995714	9.9534832565	2.4692134075
C	-0.7038427334	-1.4992253439	14.3301889958
C	6.7153609995	10.7427541851	1.6578436079
C	0.2012045926	-2.2365121685	15.0788707114
C	7.0090030777	0.7342056690	1.2187197436
C	-0.3081060476	7.6682143718	15.5780173369
C	8.2998311874	1.1816455700	1.4600448567
C	-1.5932719351	7.2264574257	15.2984041969
C	9.1709448953	0.4120788056	2.2195964144
C	-2.4420529379	8.0050026293	14.5221938409
C	8.8075788650	10.3600898366	2.8039153596
C	-1.9874831692	-1.9850490917	14.0927587483

(V_I^+ap)

Pb	-0.0012284267	0.0054421954	0.0199158524
Pb	4.3973891481	4.3685643528	0.0230612338
Pb	5.8650097553	1.5455170770	16.3172115635
Pb	1.5584080886	5.9158279598	16.3412647008
Pb	0.0534514778	8.7446842992	0.0213600121
Pb	4.4520010319	13.1084624525	0.0211234368
Pb	5.9618982026	10.2635784363	16.3296733619
Pb	1.6204685315	14.6287472708	16.3548665672
Pb	8.7377354598	0.0047060441	0.0220276969
Pb	13.1353772220	4.3689322689	0.0206135483
Pb	14.6467805770	1.5294984137	16.3762898264
Pb	10.2963093626	5.8999222427	15.9854244522
Pb	8.7923717099	8.7444692587	0.0210420057
Pb	13.1912292034	13.1084909732	0.0220664116
Pb	14.7194880336	10.2617228434	16.3691502601
Pb	10.3659630409	14.6094193408	16.2923319703
I	6.0194405462	7.1340302139	0.0445919906
I	4.1715908263	4.5067441508	3.2386502494
I	7.7062190586	7.2881744540	29.5068572412
I	2.8475538845	2.9119418264	29.5006948822
I	9.0217051954	8.8912281628	3.2446650158
I	8.7778571146	8.6457959470	16.4300598803
I	3.1206898730	3.1219729002	16.3412425301
I	1.4278125260	6.1440579497	13.1542242909
I	5.7572367565	1.3470256821	13.1260769805
I	6.1404449105	10.4691575327	19.5464396104
I	7.1784206725	2.7894900823	0.0589527864
I	4.3489209278	7.4834517814	16.3441004680
I	7.5479122448	4.3437758931	16.3433264860
I	1.6252216578	5.9622223157	0.0088718797
I	2.7771686622	1.6061654075	-0.0349216244
I	6.0746051513	15.8740196233	0.0437767256
I	4.2258936659	13.2466271579	3.2372047535
I	7.7609840657	16.0294002256	29.5060911245
I	2.9032827916	11.6502287591	29.5049234780
I	9.0768881273	17.6333436238	3.2443550120
I	8.6984412918	17.3357058070	16.2157440717
I	3.2081069607	11.8464140910	16.3636730783
I	10.4766083664	14.4806374272	19.5407127979
I	1.4900883956	14.8769981419	13.1588200946
I	5.8379238601	10.0448072668	13.1320190967
I	6.1859752811	19.2532902622	19.5476400532
I	7.2329483214	11.5289309746	0.0586169157
I	4.3936348513	16.2521188002	16.3681149706
I	7.5770041077	13.0321130971	16.2801461220
I	1.6796440562	14.7030680428	0.0087201560
I	2.8312009113	10.3460249748	-0.0349250199
I	14.7578165072	7.1337596918	0.0448402799
I	12.9086636503	4.5069078617	3.2378475138
I	16.4430678004	7.2892759066	29.5055457999
I	11.5869581786	2.9113469742	29.5016108752
I	17.7618982161	8.8927105404	3.2449754051
I	17.4857478868	8.6989591694	16.4144810335
I	11.8059944272	3.1502895160	16.6201666377
I	19.1542898463	5.6583760987	19.5710816787
I	10.2034626283	6.0986292104	12.9955712224
I	14.4648997026	1.3662680706	13.1869040470
I	14.8299421275	10.4652731151	19.5855021453
I	15.9155439206	2.7897859180	0.0600202454
I	13.0566736726	7.4426819775	16.4953630109
I	16.2586653093	4.3007719787	16.3183730187
I	10.3605444620	5.9606587151	0.0126147614
I	11.5155255378	1.6054278787	-0.0352634076
I	14.8140462780	15.8736966062	0.0451032205
I	12.9628184157	13.2473636385	3.2396248907
I	16.4979157325	16.0302144923	29.5093505072
I	11.6418122556	11.6489349382	29.5021068974
I	17.8168202232	17.6326839428	3.2415884490
I	17.4860433128	17.4143442221	16.3738832645
I	11.9427563058	11.8492845874	16.3301337316
I	19.2213480920	14.4036419469	19.5625623390
I	10.2623519147	14.8640609070	13.1158025206
I	14.5302980186	10.0307914769	13.1710816014
I	14.9210145902	19.2372050221	19.6027392852
I	15.9713932285	11.5288958217	0.0599908918
I	13.1117258160	16.2601067759	16.4012093478
I	16.3078241834	13.0207234612	16.3137660484
I	10.4173637539	14.7020252834	0.0101009250
I	11.5697840416	10.3458535144	-0.0342136686
N	11.2197799243	8.0923764273	30.0730996412

(V_I^+ap)

Pb	-0.0014167321	0.0044168741	0.0216507372
Pb	4.3968008970	4.3675289256	0.0241806518
Pb	5.8695145148	1.5460402429	16.3503142814
Pb	1.5584742336	5.9064685941	16.3373457076
Pb	0.0533070636	8.7443895478	0.0230862731
Pb	4.4518352087	13.1074634907	0.0215825292
Pb	5.9740015910	10.2595241636	16.3498980630
Pb	1.6160100914	14.6294747765	16.3533949353
Pb	8.7376272889	0.0045006257	0.0236858630
Pb	13.1350892499	4.3680410786	0.0215301693
Pb	14.6369761484	1.5220395401	16.3819835249
Pb	10.3008350934	5.8905840088	15.9613893537
Pb	8.7924639737	8.7441092431	0.0225123939
Pb	13.1908353211	13.1075589254	0.0230578057
Pb	14.7076146271	10.2596097048	16.3693750205
Pb	10.3701054441	14.6148753436	16.3035301596
I	6.0205663776	7.1309427169	0.0449526879
I	4.1702633415	4.5039056794	3.2343408682
I	7.7077767458	7.2894151609	29.5112979872
I	2.8460922060	2.9141844789	29.5064520528
I	9.0248889574	8.8899951027	3.2417684929
I	8.7555781085	8.6597209636	16.4317348504
I	3.1509437344	3.1253277222	16.3502914567
I	1.4372539417	6.1516242581	13.1264081785
I	5.7569341735	1.3388810449	13.1288554196
I	6.1412437362	10.4792480194	19.5925950470
I	7.1761191487	2.7868250116	0.0628462774
I	4.3110473447	7.4953637018	16.3717706709
I	7.5981721802	4.3032819952	16.3765115437
I	1.6258790019	5.9633919351	0.0075412633
I	2.7764597778	1.6058129217	-0.0382790176
I	6.0752893473	15.8712402118	0.0434346101
I	4.2246139435	13.2438016602	3.2326796088
I	7.7640194684	16.0311217636	29.5102071592
I	2.9008223596	11.6520912879	29.5105100944
I	9.0800469500	17.6321362153	3.2407586279
I	8.6892046726	17.3440041101	16.2216124971
I	3.2448833924	11.8523463906	16.3455459489
I	10.4823997037	14.4801824501	19.5774701549
I	1.4866146783	14.8940325965	13.1250726379
I	5.8389052982	10.0366528209	13.1276003514
I	6.1937968345	19.2596434034	19.6148912182
I	7.2313012104	11.5266036361	0.0625144417
I	4.3431247475	16.2536846944	16.3875159275
I	7.6131323008	13.0288281589	16.3212384372
I	1.6801325734	14.7035585092	0.0081059337
I	2.8310609196	10.3459454388	-0.0369476801
I	14.7590227499	7.1306670378	0.0454801311
I	12.9072579678	4.5034989408	3.2334252797
I	16.4436297689	7.2903953245	29.5101589524
I	11.5844547214	2.9138288045	29.5066910741
I	17.7653609125	8.8915279829	3.2424123643
I	17.4408898917	8.6798303063	16.3739825573
I	11.8291339982	3.1216625805	16.6326933850
I	19.1470943608	5.6469052363	19.5940237371
I	10.1779603374	6.1214534252	12.9470411463
I	14.4559994432	1.3527857426	13.1662768749
I	14.8399871857	10.4715271829	19.6137926351
I	15.9133963620	2.7867761759	0.0637756500
I	13.0182364447	7.4687968120	16.4556752396
I	16.2928610675	4.2804683266	16.2906074563
I	10.3618860958	5.9623201482	0.0110528982
I	11.5144958529	1.6059052076	-0.0373429904
I	14.8143160349	15.8712820762	0.0449277483
I	12.9617581256	13.2442647073	3.2353392482
I	16.4991413710	16.0320412619	29.5142571709
I	11.6402854845	11.6514736594	29.5073620018
I	17.8193916262	17.6311344423	3.2392929178
I	17.4612427430	17.4211924172	16.3699793620
I	11.9563777575	11.8491179204	16.3377299527
I	19.2188337533	14.3825201602	19.5896763104
I	10.2542914857	14.8750782416	13.1044345059
I	14.5221096315	10.0255812944	13.1386716073
I	14.9283231508	19.2462652858	19.6317573580
I	15.9691569821	11.5261350833	0.0643620266
I	13.0953790650	16.2472505665	16.3976617502
I	16.3464900134	13.0195536928	16.3227763705
I	10.4191951188	14.7040871853	0.0086838459
I	11.5693121025	10.3456003984	-0.0366543227
N	11.2169857798	8.0931218549	30.0732559838

N	0.6522397303	3.7146226080	2.7221178206	N	0.6537949205	3.7139462707	2.7227599784
N	8.1090057764	3.7220864983	30.0705521966	N	8.1096663561	3.7221606310	30.0703198312
N	3.7561703508	8.0743867377	2.6416735286	N	3.7565199972	8.0743600922	2.6415550650
N	9.6160136043	1.6763444369	18.9980645064	N	9.6109576709	1.6811276850	18.9908004024
N	2.2516760087	9.6522317797	13.7073937602	N	2.2546607916	9.6511288313	13.7174452725
N	5.1699461898	5.2342100869	18.8956981375	N	5.1682368989	5.2314890211	18.9894247436
N	6.5856549620	6.5540531630	13.6286020005	N	6.6002046688	6.5619624945	13.7183800650
N	11.2751904229	16.8316203717	30.0722808263	N	11.2731218795	16.8319855373	30.0725544641
N	0.7070847020	12.4540723530	2.7222543406	N	0.7081630483	12.4537685339	2.7237168226
N	8.1639382070	12.4624527504	30.0702900833	N	8.1641586862	12.4619692200	30.0700947931
N	3.8114832347	16.8141256969	2.6418442978	N	3.8116490690	16.8138746381	2.6418907080
N	9.6647994675	11.0590708021	19.0040031864	N	9.6630358941	11.0600488956	18.9955515293
N	2.3036362132	18.3882244077	13.7035875529	N	2.3077668948	18.3862487521	13.7185085696
N	5.3127830569	13.9718675112	18.9957856559	N	5.3050698627	13.9709441365	18.9902879362
N	6.6507640017	15.2955297415	13.6271590440	N	6.6583593311	15.3047054070	13.7139552993
N	19.9586971601	8.0919208273	30.0726921762	N	19.9561082591	8.0922297006	30.0726525392
N	9.3907219053	3.7135970478	2.7229557167	N	9.3921153094	3.7134179527	2.7240435461
N	16.8481870749	3.7220665434	30.0700675079	N	16.8485717287	3.7222145815	30.0703502885
N	12.4951103889	8.0742671913	2.6415881609	N	12.4955806889	8.0742780353	2.6415137994
N	18.3569590765	2.1440451045	19.0070590789	N	18.3534449751	2.1444022845	18.9924868194
N	10.9886343809	9.6615608005	13.7039443777	N	10.9903994737	9.6569616411	13.7149234355
N	14.1933358183	5.2416992025	19.0809643785	N	14.1840917072	5.2326501136	18.9907317321
N	15.3471129824	6.5637922331	13.7061883996	N	15.3522525119	6.5676977095	13.7149397835
N	20.0136673289	16.8319947090	30.0726540545	N	20.0113036991	16.8322498058	30.0725506377
N	9.4458815405	12.4538659805	2.7225280121	N	9.4471700996	12.4539010111	2.7235027503
N	16.9028376674	12.4621721686	30.0700109328	N	16.9030460373	12.4619813169	30.0697609789
N	12.5503006215	16.8138922950	2.6416784811	N	12.5506848451	16.8137594218	2.6416546021
N	18.4149945216	10.8940700601	19.0836318563	N	18.4033602043	10.8914495591	18.9923358976
N	11.0468642779	18.3925755236	13.7131390672	N	11.0496622041	18.3909749647	13.7234368418
N	14.0551598541	13.9740998320	19.0014979304	N	14.0537794046	13.9711535604	18.9926362868
N	15.3968601721	15.3065368165	13.7076874784	N	15.4037652492	15.3068179218	13.7184034251
C	3.0516947874	7.9277896948	27.1640764007	C	3.0422605048	7.9249237853	27.1666642758
C	8.8244220333	3.8678109526	5.6292973537	C	8.8256777918	3.8638843806	5.6297513073
C	7.4021936783	3.5574674263	27.1645841239	C	7.4003198694	3.5644748208	27.1669654878
C	4.4767663628	8.2417576120	5.5470414585	C	4.4858502693	8.2357028961	5.5421501424
C	6.1530256261	6.2076436265	19.5359760130	C	6.1532835785	6.2038134748	19.5260987343
C	5.5212879009	5.6913826021	13.0456519086	C	5.5573760941	5.6827635478	13.1230061737
C	3.3552272786	7.0356117758	29.4996911114	C	3.3532326967	7.0344013700	29.5027932438
C	8.5342508556	4.7810600733	3.3020314995	C	8.5373775763	4.7827163769	3.3031699939
C	5.3608946234	5.9552659147	21.9136227409	C	5.3601625298	5.9603397916	21.9053056845
C	6.4693682732	5.9351934745	10.7296839762	C	6.4757999833	5.9362757236	10.7873493944
C	9.7698294404	10.4075558143	21.9527214150	C	9.7773763313	10.4082208612	21.9441959838
C	2.0346686122	1.5220981971	10.7869884904	C	2.0240168838	1.5427034687	10.8133731871
C	4.6892460156	4.8053268928	22.3169657478	C	4.6890204688	4.8125099356	22.3165507782
C	7.1938515360	7.0548265259	10.3302869108	C	7.1878371642	7.0565067854	10.3670389079
C	6.5283174497	5.8719464828	20.9695957126	C	6.5275114559	5.8724167674	20.9616911100
C	5.2541784958	6.0810432847	11.6030622859	C	5.2774926794	6.0806496359	11.6844054069
C	2.2995867811	10.4240284593	21.0916024761	C	2.2842825377	10.4113896633	21.0020907632
C	9.6413968203	1.3301707284	11.6446300661	C	9.6354070367	1.3608331586	11.6687035353
C	3.8272637685	7.2792064331	23.2297200373	C	3.8130407038	7.2931836815	23.1963331209
C	8.0399986405	4.5324113073	9.5426976944	C	8.0237236640	4.5347202263	9.5677832980
C	8.0089479140	2.5408812798	23.2617054941	C	8.0151756386	2.5416292033	23.2904439784
C	3.8470743647	9.2619497832	9.4692190510	C	3.8346290237	9.2674390975	9.4816393635
C	7.1957251410	2.6857298486	29.5199347526	C	7.1976016444	2.6842584165	29.5197794041
C	4.6795071221	9.1046133488	3.1876651145	C	4.6895311678	9.0987222017	3.1836463717
C	2.0036636368	10.0301498471	19.6557017165	C	1.9788936772	10.0155328889	19.5680936372
C	9.8914343112	1.7611684748	13.0789246206	C	9.9010126107	1.7766994286	13.1047337106
C	4.2882416237	8.5118471234	26.8921022589	C	4.2740367949	8.5167113976	26.8905321962
C	7.5777343048	3.2981570871	5.8842709714	C	7.5787813765	3.2946633025	5.8846790861
C	9.3306107883	9.1195462904	22.2531202295	C	9.3339151774	9.1179714649	22.2284604499
C	2.5330173285	2.7834520403	10.4661855011	C	2.5409490114	2.7996980556	10.5055717002
C	6.1426797601	4.0449535103	26.8161273264	C	6.1404123204	4.0468745717	26.8126721772
C	5.7357258201	7.7537754837	5.8975446461	C	5.7429868954	7.7496030226	5.9017473006
C	2.9604711791	6.7283849323	28.0662668372	C	2.9597119691	6.7251964264	28.0692758863
C	8.9374512502	5.0724062174	4.7363134698	C	8.9394636348	5.0698864245	4.7386780511
C	10.6235538139	8.4864213136	26.6185766887	C	10.6223930791	8.4823426111	26.6200942393
C	1.2143866248	3.2873601036	6.1776216460	C	1.2143422311	3.2916622292	6.1845906856
C	7.1491173315	5.8118931928	25.5314924467	C	7.1458914531	5.8209449994	25.5363141640
C	4.7255760176	5.9955827547	7.1909470215	C	4.7261875748	5.9881509275	7.1856211003
C	3.2204620707	10.2105225941	25.5610793502	C	3.1904408574	10.2038861790	25.5562899691
C	8.6097478221	1.5745150172	7.2103762691	C	8.6097073573	1.5747502614	7.2167720792
C	7.5929199704	11.1081967472	28.0762053602	C	7.5885028556	11.1096554287	28.0770736333
C	4.2939594740	0.6872460225	4.6314757585	C	4.3071171476	0.6823361747	4.6301034634
C	8.5313997784	4.2143389227	26.6844582665	C	8.5291460896	4.2279994293	26.6950392941
C	3.3462566436	7.5902529269	6.0312273130	C	3.3527827380	7.5806237361	6.0155561764
C	4.9168918578	7.1935626050	22.3749900133	C	4.9096735921	7.2020772056	22.3513723556
C	6.9080668558	4.6723130828	10.3327202456	C	6.9090352280	4.6735215482	10.3826061342
C	8.4059951250	5.3387144130	25.8772089363	C	8.4028348127	5.3532523922	25.8888294424
C	3.4700399636	6.4695284676	6.8434426749	C	3.4725299613	6.4591526692	6.8276227034
C	6.0157394001	5.1586533894	25.9987832915	C	6.0129835499	5.1618347725	25.9966255895
C	5.8601404401	6.6442585594	6.7209522770	C	5.8631318958	6.6389042601	6.7243952769
C	3.1549124332	6.1264122031	23.6149567500	C	3.1430559883	6.1424275550	23.5915627527
C	8.7614139051	5.6558004728	9.1582174457	C	8.7325192301	5.6588417621	9.1630068838
C	9.1394594029	2.6529307618	22.4595421600	C	9.1441625264	2.6482338833	22.4854242475
C	2.7250011325	9.1469371037	10.2829783684	C	2.7081279610	9.1553845659	10.2901760806
C	3.5846359998	4.8912586815	23.1554238781	C	3.5793423137	4.9037558380	23.1480954250
C	8.3390749526	6.9152572147	9.5551563476	C	8.3154325507	6.9178684404	9.5663068610
C	7.5174255947	10.0120511968	23.5570606799	C	7.5207861024	9.9989714215	23.5405360554
C	4.2886041408	1.7817976374	9.1568354959	C	4.2874128802	1.7852468045	9.1933296093
C	8.2115292242	8.9224724866	23.0463448158	C	8.2119354112	8.9142106879	23.0158849080
C	3.6517672183	2.9102119018	9.6577596752	C	3.6649997058	2.9177645053	9.7031147866
C	4.3698335650	9.6476136886	26.1012261593	C	4.3447252931	9.6512259238	26.0963165559
C	7.4734504209	2.1564292217	6.6630907958	C	7.4739603994	2.1544473187	6.6660036528
C	10.6922350886	9.6149368533	25.8091156768	C	10.6893464932	9.6083466826	25.8065859599
C	1.1086213897	2.1462224324	6.9657721689	C	1.1092505077	2.1534161455	6.9772783542
C	3.0944603440	16.6631858710	27.1622605502	C	3.0915074908	16.6627044789	27.1647406341
C	8.8760582744	12.6175026981	5.6228971244	C	8.8758023303	12.6215966646	5.6219881532
C	7.4518358790	12.2944151350	27.1635911524	C	7.4477831384	12.2985540625	27.1680865193
C	4.5340775307	16.9817248814	5.5469197751	C	4.5426566214	16.9743968816	5.5429665332
C	6.3558637518	14.8496588531	19.5898873988	C	6.3373801210	14.8561102742	19.5890405018
C	5.5948201323	14.4337815681	13.0309644932	C	5.6213750473	14.4300282424	13.1038064701
C	3.4124471136	15.7776226694	29.4994456038	C	3.4078889573	15.7743642696	29.5018070395
C	8.5891020046	13.5251737974	3.2942632439	C	8.5903200746	13.5275343313	3.2918033937
C	5.4407758422	14.6026298203	21.9283138767	C	5.4326935884	14.6013788102	21.9295568386
C	6.5510159876	14.6808693389	10.7179785212	C	6.5588908120	14.6809106974	10.7747970031
C	9.8918248299	19.0016439829	21.9632982575	C	9.8791959306	18.9953997103	21.9701291102
C	2.0948832270	10.2780973444	10.7961858739	C	2.0878742943	10.2876573479	10.8132055144
C	4.7279348703	13.4835276389	22.3480916160	C	4.7165402499	13.4816363660	22.3426300627
C	7.2812205269	15.7974737955	10.3217719687	C	7.2889680928	15.7971767064	10.3761310180
C	6.6366356783	14.4558302174	21.0287341840	C	6.6257219366	14.4584792314	21.0263539293
C	5.3350603358	14.8302347611	11.5889153167	C	5.3549282654	14.8293536298	11.6626172055
C	2.3374470063	19.1127918456	21.0190926615	C	2.3461682750	19.1042857286	20.9921765072
C	9.6532479230	10.1823200418	11.6853064859	C	9.6442873624	10.1901859286	11.7064716349
C	3.9101020989	16.0046984148	23.1672836333	C	3.8901226728	16.0019399942	23.1555720866
C	8.1187981319	13.2730583400	9.5338853770	C	8.1073132366	13.2755486853	9.5611631842
C	7.9540383390	11.2988673790	23.2797481173	C	7.9613571624	11.2878768555	23.2793026706
C	3.9059756662	18.0010521048	9.4610632347	C	3.8864638278	18.0088240693	9.4852229243
C	7.2512804965	11.4249075945	29.5210093121	C	7.2500268327	11.4235592708	29.5234876943
C	4.7380475019	17.8421518471	3.1866149307	C	4.7459450115	17.8371207405	3.1839038239

C	2.0368477448	18.7359358561	19.5795761174	C	2.0325353466	18.7304468694	19.5540446604
C	9.9437863983	10.5534395135	13.1279357287	C	9.9474751061	10.5553191219	13.1490067297
C	4.3248987014	17.2563024191	26.8833246662	C	4.3215131997	17.2583868056	26.8897385505
C	7.6310986050	12.0406817195	5.8697815270	C	7.6317360341	12.0423496030	5.8676624704
C	9.3829774868	17.7455082362	22.2887479343	C	9.3525606938	17.7426192174	22.2804381520
C	2.6076834304	11.5345534055	10.4798200972	C	2.6154323641	11.5420692013	10.5127591972
C	6.1900309354	12.7809707359	26.8218402369	C	6.1860935323	12.7779216244	26.8156071075
C	5.7922087980	16.4932056214	5.8997891813	C	5.7994409293	16.4904370829	5.9068528265
C	3.0146591223	15.4656474938	28.0680273939	C	3.0115717108	15.4643048833	28.0693722466
C	8.9879323578	13.8209943707	4.7288360124	C	8.9869834008	13.8242836580	4.7268890669
C	10.6816788725	17.2290217971	26.6205881404	C	10.6816896586	17.2232328922	26.6239359018
C	1.2695800483	12.0336508880	6.1815572217	C	1.2713614869	12.0314237381	6.1871733204
C	7.1889709675	14.5391569894	25.5201974380	C	7.1863764087	14.5463188431	25.5287878760
C	4.7787953040	14.7370945319	7.1931952552	C	4.7814794439	14.7288871329	7.1892477179
C	3.2369833468	18.9367507210	25.5442447678	C	3.2342658621	18.9348918339	25.5449362176
C	8.6648487175	10.3240998650	7.2028778799	C	8.6668088409	10.3337341311	7.2098457673
C	7.6477202816	19.8487033506	28.0750497349	C	7.6474873425	19.8525409616	28.0730639920
C	4.3437197642	9.4273777485	4.6325955064	C	4.3592212980	9.4233247529	4.6295867265
C	8.5781770959	12.9480056137	26.6723174212	C	8.5747116452	12.9620976593	26.6918734713
C	3.4022682357	16.3316503310	6.0300242859	C	3.4091212729	16.3184466264	6.0141222501
C	5.0161820983	15.8650451471	22.3413306652	C	5.0052378146	15.8632669823	22.3417629939
C	6.9848609191	13.4168551855	10.3203124931	C	6.9831948208	13.4180406719	10.3621761652
C	8.4477012728	14.0676879994	25.8595341100	C	8.4446240689	14.0834769362	25.8812791603
C	3.5240203939	15.2116802629	6.8436595283	C	3.5281021318	15.1978291355	6.8274852080
C	6.0583905016	13.8901050295	25.9991246110	C	6.0552583016	13.8890737797	25.9950368049
C	5.9147384482	15.3843553981	6.7244346360	C	5.9191296218	15.3809696124	6.7314343073
C	3.2020073599	14.8813045705	23.5748221434	C	3.1758207020	14.8783491746	23.5514096333
C	8.8465386899	14.3934984594	9.1537564405	C	8.8354557604	14.3953017202	9.1797174529
C	9.0726825737	11.4921301507	22.4764236426	C	9.0815038844	11.4880263051	22.4797536601
C	2.7866642253	17.8761086030	10.2769731612	C	2.7620436271	17.8920630924	10.2958761790
C	3.6092624800	13.6228240456	23.1604836779	C	3.5882823727	13.6200221479	23.1420654051
C	8.4290373858	15.6539678559	9.5511925579	C	8.4275753794	15.6543042360	9.5917399118
C	7.6123443202	18.7653378948	23.5633161477	C	7.6024943154	18.7694462978	23.5778323405
C	4.3550004657	10.5176456911	9.1706719069	C	4.3566029607	10.5208370238	9.1978984399
C	8.2503060427	17.6305832243	23.0788067587	C	8.2205664352	17.6329809413	23.0728785306
C	3.7298078328	11.6513023347	9.6744924598	C	3.7420260436	11.6553288116	9.7128535573
C	4.3927212171	18.3882588504	26.0854620630	C	4.3891279983	18.3902172020	26.0914663754
C	7.5283659421	10.8987081757	6.6484360578	C	7.5302051577	10.9023622673	6.6497237761
C	10.7500927841	18.3585700691	25.8128312058	C	10.7445067070	18.3516247034	25.8136600924
C	1.1653371221	10.8960091341	6.9748256762	C	1.1675388321	10.8960279491	6.9840951695
C	11.7766353049	7.9230429676	27.1597756067	C	11.7761084598	7.9217574464	27.1629187701
C	17.5604849366	3.8758115440	5.6192488178	C	17.5610570630	3.8742043929	5.6189452023
C	16.1324069832	3.5565773230	27.1644295147	C	16.1307013389	3.5625442849	27.1676642088
C	13.2127874146	8.2414915549	5.5479871499	C	13.2206563283	8.2344904941	5.5431943125
C	15.2258337691	6.1525221773	19.6474415473	C	15.2037020859	6.1456639964	19.5756014053
C	14.3193884207	5.6842562891	13.0897605103	C	14.3290667414	5.6812593162	13.1037692580
C	12.0955857501	7.0386490891	29.4977188041	C	12.0936197638	7.0375574884	29.5021133340
C	17.2766014624	4.7886764006	3.2934345283	C	17.2789946165	4.7896671799	3.2931155462
C	14.2230433859	5.9377219848	21.9453364618	C	14.2169794962	5.9211948416	21.8847297411
C	15.2578930820	5.9371075247	10.7618172347	C	15.2538665837	5.9333684596	10.7693232231
C	18.5796303780	10.2881695368	21.9900040553	C	18.5754918684	10.2782423478	21.9160646086
C	10.8518335416	1.4709037802	10.7638576516	C	10.8404228247	1.4972674704	10.7800182091
C	13.5094099180	4.7993102287	22.3157475955	C	13.5007252012	4.7861147100	22.2602428660
C	15.9749932476	7.0569284653	10.3519108066	C	15.9734279560	7.0531593645	10.3623924047
C	15.4682238375	5.8178951862	21.1087357936	C	15.4511722298	5.7973494693	21.0332405123
C	14.0519742939	6.0805844971	11.6480263058	C	14.0532626415	6.0757832203	11.6626554707
C	10.9666000605	10.6114414891	21.0667926318	C	10.9705105097	10.6203005615	21.0555518324
C	18.3176169109	1.3884267181	11.6899857036	C	18.3102807028	1.4182752483	11.7212430088
C	12.6000879382	7.2944038928	23.1175030370	C	12.6163303317	7.2834052587	23.0813892049
C	16.8067346003	4.5356328453	9.5455691734	C	16.8024920484	4.5332195403	9.5504436068
C	16.7655361708	2.5359099393	23.2565115045	C	16.7912981288	2.5320071751	23.2494921155
C	12.5975200807	9.2608644712	9.4593693642	C	12.5755817002	9.2641343941	9.4640409545
C	15.9366657108	2.6835079712	29.5206038649	C	15.9355292032	2.6839749922	29.5218181591
C	13.4160136413	9.1057621096	3.1891686216	C	13.4250678769	9.1007433111	3.1857322286
C	10.7222913171	10.2165377152	19.6213330441	C	10.7250421770	10.2232749527	19.6105631738
C	18.6149575013	1.7816455380	13.1255993479	C	18.6228963636	1.7940456996	13.1591690173
C	13.0083771350	8.5119204651	26.8771218324	C	13.0071512826	8.5111318882	26.8779484411
C	16.3139054868	3.3010516390	5.8621178257	C	16.3153567916	3.2963061152	5.8587931969
C	18.0881116127	9.0254361147	22.3165065051	C	18.0803142663	9.0173616153	22.2453598811
C	11.3435454785	2.7371813104	10.4535545718	C	11.3429661561	2.7608082387	10.4742570914
C	14.8695494965	4.0413346007	26.8237538640	C	14.8684158505	4.0422974878	26.8179321540
C	14.4722478555	7.7570366554	5.9014780543	C	14.4782970584	7.7513292687	5.9049976831
C	11.6950650454	6.7265602018	28.0669898827	C	11.6961825637	6.7253283606	28.0703716356
C	17.6753916867	5.0815283941	4.7286638678	C	17.6759547570	5.0806564305	4.7293145370
C	19.3831659055	8.4981939198	26.6170668301	C	19.3688803974	8.4858054330	26.6198659319
C	9.9583522794	3.2773621345	6.1817293711	C	9.9589167948	3.2752663492	6.1854306456
C	15.8653168067	5.8078654999	25.5302818199	C	15.8664659498	5.8127077366	25.5308232599
C	13.4643197415	5.9959438281	7.1924067733	C	13.4635068101	5.9854911872	7.1839145050
C	11.9221544983	10.1996025859	25.5453518420	C	11.9187027327	10.1928642856	25.5404299183
C	17.3421197762	1.5749973598	7.1877032638	C	17.3437971067	1.5784382823	7.1948469140
C	16.3279174665	11.1094764276	28.0755091999	C	16.3256669552	11.1112130139	28.0754978881
C	13.0281500135	0.6864483923	4.6326077933	C	13.0443230496	0.6825221825	4.6295413205
C	17.2574755366	4.2142641617	26.6758246367	C	17.2566817006	4.2259911742	26.6889713611
C	12.0829774316	7.5867075276	6.0294614729	C	12.0882326637	7.5762439088	6.0138177862
C	13.7510868231	7.1872619106	22.3482923796	C	13.7569475391	7.1725368450	22.2970762772
C	15.6879492999	4.6749401431	10.3541052704	C	15.6827839244	4.6716596809	10.3581691434
C	17.1247396301	5.3372392367	25.8681086201	C	17.1251391942	5.3481455290	25.8797280167
C	12.2081291943	6.4660756202	6.8416423489	C	12.2091806318	6.4538631050	6.8244848828
C	14.7361668419	5.1537845336	26.0057472688	C	14.7366448827	5.1543489828	25.9986368386
C	14.5982528857	6.6479563470	6.7253375084	C	14.6000187241	6.6401189473	6.7269306908
C	11.8926603351	6.1529519452	23.4762458169	C	11.9068608315	6.1449579658	23.4461304719
C	17.5230255259	5.6591932360	9.1530480487	C	17.5204724454	5.6565593079	9.1607467550
C	17.8906531831	2.6477886931	22.4462875712	C	17.9153339167	2.6414606709	22.4370971026
C	11.4697664996	9.1558231586	10.2662816446	C	11.4465813646	9.1617100620	10.2703090975
C	12.3466170688	4.9059885869	23.0712669956	C	12.3482372601	4.8969307048	23.0308935835
C	17.1079354692	6.9177900392	9.5590079663	C	17.1071027639	6.9145390528	9.5702633081
C	16.3153567929	10.0245388431	23.6050540875	C	16.3333321696	10.0213138304	23.5649026694
C	13.1273860238	1.7594205435	9.1691537645	C	13.1188333233	1.7718351475	9.1856153660
C	16.9647557058	8.8970540026	23.1182785782	C	16.9673279636	8.8920235165	23.0624516176
C	12.4742842512	2.8803542473	9.6653703907	C	12.4756119770	2.8961016518	9.6873502145
C	13.0777133965	9.6449048631	26.0807951132	C	13.0751334825	9.6416667442	26.0775952221
C	16.2079015167	2.1555168033	6.6348881674	C	16.2099339449	2.1530156857	6.6352884737
C	19.4643404598	9.6298674997	25.8131114905	C	19.4389886900	9.6152499976	25.8116206558
C	9.8546305229	2.1401320431	6.9761045264	C	9.8546828459	2.1399511539	6.9828029071
C	11.8349789463	16.6653578914	27.1604936604	C	11.8375059835	16.6661645540	27.1653845156
C	17.6147806124	12.6184869115	5.6213142997	C	17.6168289901	12.6142357495	5.6243697474
C	16.1903275271	12.2969823458	27.1641148025	C	16.1870890103	12.3012937055	27.1676535227
C	13.2711188998	16.9812525041	5.5481259877	C	13.2806880603	16.9751876705	5.5430252135
C	15.1029808181	14.8432328280	19.6035281602	C	15.0934955875	14.8459637460	19.5978243838
C	14.3591312667	14.4318489289	13.0973499309	C	14.3729322758	14.4246281135	13.1113629187
C	12.1520467533	15.7782567753	29.4971007759	C	12.1547382295	15.7794698902	29.5027091187
C	17.3293369935	13.5266306603	3.2931467350	C	17.3313331110	13.5266922657	3.2963477661
C	14.1731681979	14.5982738501	21.9357335443	C	14.1677841792	14.5988545732	21.9327526985
C	15.2952226961	14.6835668846	10.7696973394	C	15.2936008159	14.6799267136	10.7773547260
C	18.6160469477	18.9950814762	21.9184540631	C	18.6327330395	18.9878798611	21.9015503644
C	10.8603402600	10.2908015895	10.7949598831	C	10.8438469212	10.2973420526	10.8059918715
C	13.4542479823	13.4820043334	22.3525736270	C	13.4425561573	13.4831689189	22.3412445881
C	16.0143072180	15.8012506659	10.3574950312	C	16.0120121825	15.7984773722	10.3651680775
C	15.3734934285	14.4465732586	21.0435395477	C	15.3661531085	14.4481276728	21.0379944346

C	14.0917542498	14.8320194132	11.6576335595	C	14.0954896551	14.8256669174	11.6731340807
C	11.0935267382	19.1178785798	21.0655324948	C	11.0774181960	19.1072966717	21.0665359847
C	18.3757966445	10.1565893680	11.6983267178	C	18.3711869000	10.1685975801	11.7181124982
C	12.6427947761	16.0061598817	23.1679167943	C	12.6317592143	16.0061504427	23.1593140795
C	16.8406989963	13.2778293589	9.5539354418	C	16.8429770806	13.2766561072	9.5628091596
C	16.7997438712	11.2859943699	23.2922920687	C	16.8204607757	11.2811121146	23.2504003697
C	12.7485020984	17.9733683862	9.4580737250	C	12.7298640441	17.9889550102	9.4699304175
C	15.9880698968	11.4262939253	29.5208339687	C	15.9881077461	11.4247283269	29.5222523788
C	13.4747407949	17.8429018001	3.1880505855	C	13.4845845533	17.8372710742	3.1837526105
C	10.8601103907	18.4967585722	19.7017100792	C	10.8428745165	18.4907069121	19.7004464252
C	18.6784546626	10.5369714124	13.1364974983	C	18.6816289326	10.5410560553	13.1572830875
C	13.0665829355	17.2540523575	26.8771044020	C	13.0663106028	17.2603207454	26.8812240530
C	16.3689124403	12.0434028679	5.8673736641	C	16.3711925669	12.0388089496	5.8708367584
C	18.0865287044	17.7411564638	22.2168694679	C	18.0969107943	17.7354409346	22.1959581647
C	11.4002666060	11.5414469647	10.5021341650	C	11.3909562656	11.5460894976	10.5172623279
C	14.9300615727	12.7824600143	26.8153760086	C	14.9265593056	12.7808878345	26.8114435072
C	14.5300125865	16.4933493115	5.8989866208	C	14.5376856487	16.4888426792	5.9029624376
C	11.7542362851	15.4671984487	28.0653502721	C	11.7627533802	15.4669642211	28.0694527836
C	17.7282590874	13.8221656719	4.7277344237	C	17.7304334281	13.8186747365	4.7316585953
C	19.4192693054	17.2208287064	26.6158612670	C	19.4169694646	17.2171680221	26.6136481020
C	10.0099600698	12.0349052700	6.1831984033	C	10.0096797258	12.0441743551	6.1874544080
C	15.9347134629	14.5449999274	25.5235404130	C	15.9306810863	14.5516024729	25.5300076099
C	13.5198261131	14.7378701885	7.1959864073	C	13.5201528064	14.7304309274	7.1900742245
C	11.9797434002	18.9434815501	25.5488399526	C	11.9714010159	18.9422595291	25.5499151469
C	17.3995951827	10.3241569492	7.1998173477	C	17.4020069047	10.3241880500	7.2093300195
C	16.3856745705	19.8485362256	28.0746135949	C	16.3829056249	19.8519418048	28.0746452889
C	13.0780124059	9.4271797358	4.6338737178	C	13.0922305530	9.4227934015	4.6317333634
C	17.3187846876	12.9528606235	26.6810140252	C	17.3153661158	12.9647731758	26.6945447693
C	12.1405848235	16.3309093715	6.0338320180	C	12.1473423504	16.3216700610	6.0178324844
C	13.7513123715	15.8624332280	22.3460948543	C	13.7473870261	15.8625248525	22.3469249053
C	15.7234668516	13.4201100704	10.3639536838	C	15.7237737770	13.4170980557	10.3707633425
C	17.1918875189	14.0739659384	25.8698184366	C	17.1877351591	14.0870789752	25.8849421193
C	12.2643478602	15.2116658779	6.8480290944	C	12.2666040597	15.2015932337	6.8318458878
C	14.8019804077	13.8932769873	25.9940818820	C	14.7982321758	13.8931749946	25.9917595521
C	14.6545453947	15.3852645558	6.7244143058	C	14.6576006687	15.3796282173	6.7277851325
C	11.9272492581	14.8858897730	23.5708664326	C	11.9081623797	14.8868483416	23.5503611831
C	17.5582467915	14.3995766473	9.1583908068	C	17.5598675916	14.3990096464	9.1681091795
C	17.9248776964	11.4131603353	22.4849085674	C	17.9341931278	11.4045639119	22.4263870511
C	11.6193119963	17.8325514745	10.2579259736	C	11.5990292956	17.8558686301	10.2691165501
C	12.3311136264	13.6258334432	23.1580606380	C	12.3127589501	13.6270722059	23.1377559837
C	17.1459897674	15.6593602513	9.5634211966	C	17.1453484291	15.6580630108	9.5729501582
C	16.3500349089	18.7626149730	23.5365523494	C	16.3699890336	18.7603711111	23.5258603740
C	13.1306992487	10.5110013738	9.1825318557	C	13.1163149527	10.5122039763	9.1926748599
C	16.9610839408	17.6278004003	23.0181121137	C	16.9731544536	17.6247355431	23.0001900929
C	12.5274396044	11.6488092190	9.7028988719	C	12.5192022671	11.6506997225	9.7185031600
C	13.1360469607	18.3876334111	26.0816408512	C	13.1302925796	18.3931535775	26.0838038218
C	16.2643622155	10.9010683510	6.6452057466	C	16.2669908286	10.8979109601	6.6511143141
C	19.4865430881	18.3474098289	25.8038733289	C	19.4843142454	18.3428029177	25.7998980578
C	9.9077128601	10.8977668183	6.9774285775	C	9.9086090772	10.9100747409	6.9862334862

Bibliography

(1) Marini, A.; Hogan, C.; Grüning, M.; Varsano, D. Yambo: An Ab Initio Tool for Excited State Calculations. *Computer Physics Communications* **2009**, *180* (8), 1392–1403. https://doi.org/10.1016/j.cpc.2009.02.003.

(2) Dyksik, M.; Duim, H.; Zhu, X.; Yang, Z.; Gen, M.; Kohama, Y.; Adjokatse, S.; Maude, D. K.; Loi, M. A.; Egger, D. A.; Baranowski, M.; Plochocka, P. Broad Tunability of Carrier Effective Masses in Two-Dimensional Halide Perovskites. *ACS Energy Lett.* **2020**, *5* (11), 3609–3616. https://doi.org/10.1021/acsenergylett.0c01758.

(3) Van de Walle, C. G.; Neugebauer, J. First-Principles Calculations for Defects and Impurities: Applications to III-Nitrides. *Journal of Applied Physics* **2004**, *95* (8), 3851–3879. https://doi.org/10.1063/1.1682673.

(4) Umari, P.; Pasquarello, A. Ab Initio Molecular Dynamics in a Finite Homogeneous Electric Field. *Phys. Rev. Lett.* **2002**, *89* (15), 157602. https://doi.org/10.1103/PhysRevLett.89.157602.
